# Senescent glia link mitochondrial dysfunction and lipid accumulation

**DOI:** 10.1038/s41586-024-07516-8

**Published:** 2024-06-05

**Authors:** China N. Byrns, Alexandra E. Perlegos, Karl N. Miller, Zhecheng Jin, Faith R. Carranza, Palak Manchandra, Connor H. Beveridge, Caitlin E. Randolph, V. Sai Chaluvadi, Shirley L. Zhang, Ananth R. Srinivasan, F. C. Bennett, Amita Sehgal, Peter D. Adams, Gaurav Chopra, Nancy M. Bonini

**Affiliations:** 1 Medical Scientist Training Program, Perelman School of Medicine, University of Pennsylvania, Philadelphia, PA USA; 2 Department of Biology, University of Pennsylvania, Philadelphia, PA USA; 3 Neuroscience Graduate Group, Perelman School of Medicine, University of Pennsylvania, Philadelphia, PA USA; 4 Cancer Genome and Epigenetics Program, Sanford Burnham Prebys Medical Discovery Institute, La Jolla, CA USA; 5 Department of Chemistry, Purdue University, West Lafayette, IN USA; 6 Howard Hughes Medical Institute and Chronobiology and Sleep Institute, Perelman School of Medicine at the University of Pennsylvania, Philadelphia, PA USA; 7 Department of Psychiatry, Perelman School of Medicine, University of Pennsylvania, Philadelphia, PA USA; 8 Division of Neurology, Children’s Hospital of Philadelphia, Philadelphia, PA USA; 9 Purdue Institute for Integrative Neuroscience, Purdue University, West Lafayette, IN USA; 10 Purdue Institute for Drug Discovery, Purdue University, West Lafayette, IN USA; 11 Purdue Center for Cancer Research, Purdue University, West Lafayette, IN USA; 12 Purdue Institute of Inflammation, Immunology and Infectious Disease, Purdue University, West Lafayette, IN USA

**Keywords:** Cellular neuroscience, Glial biology, Neural ageing, Senescence

## Abstract

Senescence is a cellular state linked to ageing and age-onset disease across many mammalian species^1,2^. Acutely, senescent cells promote wound healing^3,4^ and prevent tumour formation^5^; but they are also pro-inflammatory, thus chronically exacerbate tissue decline. Whereas senescent cells are active targets for anti-ageing therapy^6–11^, why these cells form in vivo, how they affect tissue ageing and the effect of their elimination remain unclear^12,13^. Here we identify naturally occurring senescent glia in ageing *Drosophila* brains and decipher their origin and influence. Using Activator protein 1 (AP1) activity to screen for senescence^14,15^, we determine that senescent glia can appear in response to neuronal mitochondrial dysfunction. In turn, senescent glia promote lipid accumulation in non-senescent glia; similar effects are seen in senescent human fibroblasts in culture. Targeting AP1 activity in senescent glia mitigates senescence biomarkers, extends fly lifespan and health span, and prevents lipid accumulation. However, these benefits come at the cost of increased oxidative damage in the brain, and neuronal mitochondrial function remains poor. Altogether, our results map the trajectory of naturally occurring senescent glia in vivo and indicate that these cells link key ageing phenomena: mitochondrial dysfunction and lipid accumulation.

## Abstract (web summary)

The authors identify naturally occurring senescent glia in aged *Drosophila* brains and decipher their origin and influence, determining that they can appear in response to neuronal mitochondrial dysfunction and that they promote lipid accumulation, indicating that these cells link key ageing phenomena.

## Main

Across mammalian species and tissues, ageing is associated with the onset of cellular senescence: an inflammatory secretory state adopted by a minority cell population. Acutely, senescent cells are thought to promote wound recovery and aid in tumour suppression^3,4^ but seem to be deleterious chronically^16^. Indeed, periodically eliminating senescent cells in ageing mice can extend life, improve tissue health and mitigate age-onset disease^8–11,17^. These benefits are attributed to reduced senescence-associated secretory phenotype (SASP) and associated inflammation. Yet, fundamental questions about senescent cells remain to be addressed. Mechanistic insight largely comes from in vitro studies or animal models in which senescence is induced^13^. Whether such models capture what happens naturally is unclear, as identifying and manipulating naturally occurring senescent cells in animals is challenging. Overall, how and why cells senesce in vivo, as well as their effect on tissue ageing, largely remains a mystery.

In the *Drosophila* brain, we previously found the senescence-associated transcription factor AP1 (refs. ^14,18^) is chronically active in a subset of glia after traumatic brain injury and in advanced age^15^. These AP1^+^ glia have an abnormal morphology, produce matrix metalloproteinase and promote tau pathology^15^, which are traits of senescent glia in mice^8^. Whereas senescent cells have not been reported in the fly^19^, senescence-associated genes are conserved and fly cells can undergo oncogene-induced senescence in vivo^20,21^. Here, we identify naturally occurring senescent-like glia in *Drosophila*, and study their origin and impact in vivo. In ageing fly brains, AP1 becomes active in a subset of glia that show traits and biomarkers similar to senescent cells in mammals. We determine that these senescent AP1^+^ glia appear in response to neuronal mitochondrial dysfunction and are associated with lipid droplet (LD) accumulation in non-senescent glia. Similarly, we find that AP1 activity in human senescent fibroblasts promotes LDs in non-senescent fibroblasts in vitro. Reducing AP1 activity in glia prevents hallmarks of senescence, with both beneficial and detrimental effects to the ageing brain.

## AP1^+^ glia have a senescent phenotype

**Fig. 1:**
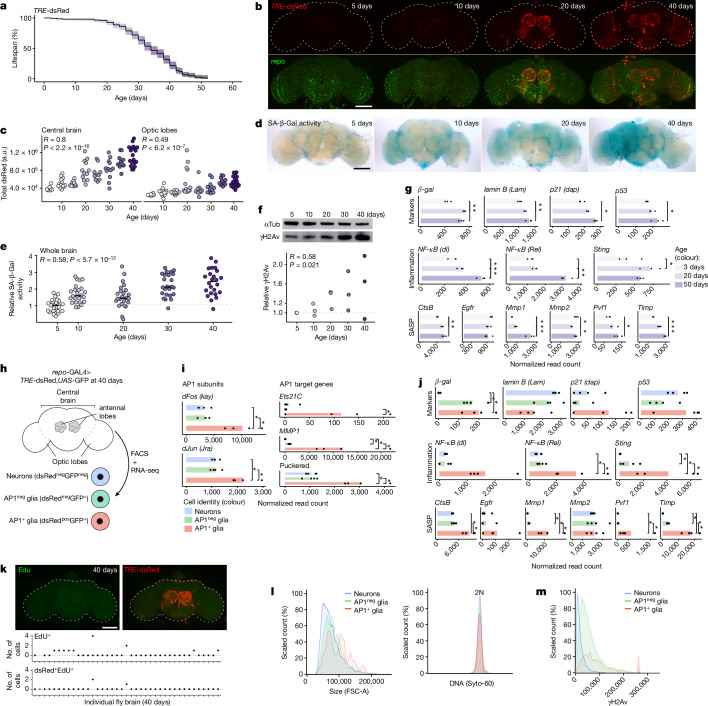
AP1^+^ glia appear with age and have a senescent phenotype. **a**, Lifespan of genomic AP1 reporter line (*TRE*-dsRed; *n* = 200 flies). **b**, Representative images of fly brains showing age-onset AP1 activity (dsRed; top) is mostly in glial cells (repo; bottom). **c**, Quantification of dsRed intensity shows that AP1 activity is higher the central brain (left) versus optic lobes (right) (*TRE*-dsRed; *n* = 93 brains). See Extended Data Fig. 1a–c for high-magnification images and quantification of dsRed colocalization with glial versus neuronal markers. a.u., arbitrary units. **d**,**e**, Representative images showing SA-β-Gal activity increases with age (**d**) with quantification (**e**) (*TRE*-dsRed; *n* = 119 brains). **f**, Brain γH2Av levels increase with age (*TRE*-dsRed; *n* = 8 brains per replicate). For gel source data, see Supplementary Fig. 1a. **g**, Bulk RNA-seq of brains shows that senescence-associated genes increase with age (*w*^*1118*^; *n* = 20 brains per replicate). **h**, Cells were FACS-isolated from 40-day-old brains for bulk RNA-seq (*repo*-GAL4 > *TRE*-dsRed;*UAS*-GFP; *n* = 500 cells per replicate). **i**,**j**, Expression of AP1 subunits (*dFos*, *dJun*), AP1-target genes (**i**) and senescence-associated genes (**j**) is highest in AP1^+^ glia. See Supplementary Data 1 for differential expression genes. **k**, Most AP1^+^ glia are non-dividing by EdU labelling at 40 days (*TRE*-dsRed; *n* = 39 brains). **l**, Analysis of live FACS-isolated cells from 40-day-old brains shows that AP1^+^ glia are larger (left) with normal DNA content (right) (*n* = 4,748 neurons, *n* = 14,326 AP1^neg^ glia, *n* = 490 AP1^+^ glia). **m**, Distribution of γH2Av staining in fixed FACS-isolated cells from 40-day-old brains (*n* = 8,609 neurons, *n* = 845 AP1^neg^ glia, *n* = 302 AP1^+^ glia). For all bar graphs, data shown are means. Each point in a microscopy experiment represents one brain; in immunoblot or bulk RNA-seq experiments it represents one biological replicate. All data were collected from two or three independent experiments. Pearson’s correlation (**c**,**e**,**f**). Precise *n* and *P* values are in the Source Data. *P-adjusted < 0.05 for sequencing data; ***P < 0.001; **P < 0.01, *P < 0.05 for all other data. All scale bars, 100 μm. Source Data

**Extended Data Fig. 1:**
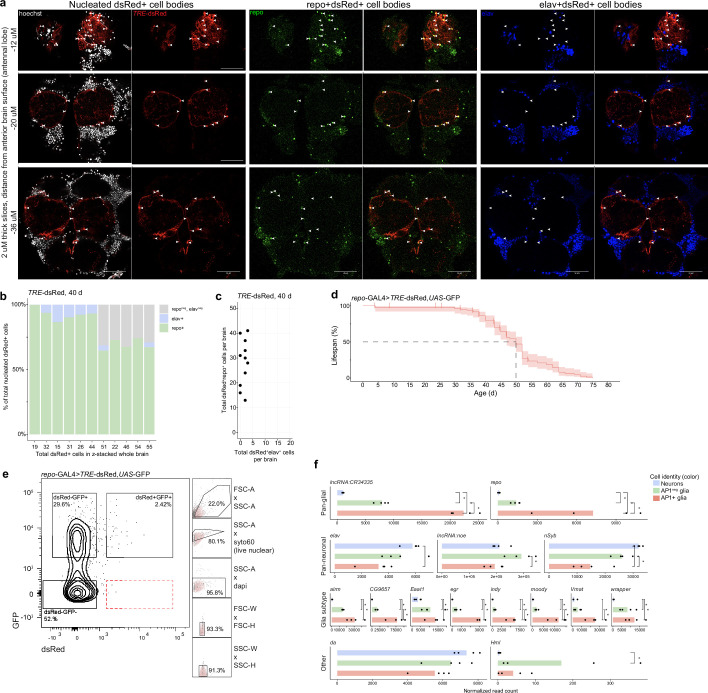
AP1 is active in a subset of glia with age. **a**, Representatives high magnification images of a 40 d brain showing the co-localization of AP1 activity (dsRed) with glial (repo) or neuronal (elav) markers. **b**, Proportion of dsRed+ cells in 40 d brains (x-axis shows total number) positive for repo (green), elav (blue) or neither (grey), with the total number of dsRed+repo+ and dsRed+elav+ cells shown in (**c**). **d**, Lifespan of genotype used for FACS-isolation of neurons, AP1^neg^ glia, and AP1+ glia (*n* = 100 flies). **e**, Representative gating strategy used for FACS-based isolation of neurons (dsRed^neg^GFP^neg^), AP1^neg^ glia (dsRed^neg^GFP + ) and AP1+ glia (dsRed+GFP + ) for bulk RNA-sequencing. **f**, Expression of neuronal and glial marker genes in FACS-isolated and bulk RNA-sequenced neurons, AP1^neg^ glia, and AP1+ glia (*repo*-GAL4 > *TRE*-dsRed,*UAS*-GFP; *n* = 500 cells per replicate). For all bar graphs, data are mean. Each point in a microscopy experiment represents one brain and in bulk RNA-sequencing experiments it depicts one biological replicate. All data were collected from two or three independent experiments. Precise *n* and *P* values are provided in the Source Data. *P-adjusted<0.05 for sequencing data; ***P < 0.001; **P < 0.01, *P < 0.05 for all other data. Source Data

As our previous work suggested AP1^+^ glia have senescent features^15^, we used a transgenic line with dsRed expressed under the control of an AP1 binding motif (*TRE-*dsRed)^22^ to characterize when and where AP1^+^ glia appear in the brain. In early life (5–10 days; lifespan, Fig. 1a), there were no dsRed^+^ cells (Fig. 1b,c). By mid-life (roughly 20 days), dsRed^+^ cells appeared in the antennal lobes (diagram, Fig. 1h), which comprise neurons known to degenerate in early life^23^. In late life (30–40 days), dsRed^+^ cells persisted in the antennal lobes and also appeared in the optic lobes. Costaining with nuclear glial (repo) and neuronal (elav) antibodies showed most dsRed^+^ cells are repo^+^ (64–100%; Extended Data Fig. 1a–c), consistent with our previous work^15^. We asked whether biomarkers associated with senescence coincide with the appearance of AP1^+^ glia in age. Senescence-associated β-galactosidase (SA-β-Gal) activity^24^ (Fig. 1d,e) and the fly DNA damage marker, γH2Av (ref. ^25^), increased with age (Fig. 1f). Analysis of bulk RNA-sequencing (RNA-seq) data from wild-type fly brains (3, 20, 50 days; previously published^26^) indicated genes commonly associated with senescence increase with age, including *Gal* (β-Gal), *dacapo* (fly p21 (ref. ^27^)), inflammatory transcription factors (fly NF-κβs^28^, *Sting*^29^) and orthologues of SASP genes defined in mammals^5^ (Fig. 1g). Thus, as fly brains age, AP1^+^ glia accumulate in a stereotyped regionally progressive manner and markers of cellular senescence increase.

To determine whether AP1^+^ glia are the source of senescence biomarkers, we used fluorescence-activated cell sorting (FACS) followed by bulk RNA-seq to characterize select cell populations from aged brains (40 days). Glia were tagged with green fluorescent protein (GFP) expressed under a constitutively active, glia-specific GAL4 and AP1 activity was identified by dsRed (*repo-*GAL4>*TRE-*dsRed*,UAS-*GFP; lifespan in Extended Data Fig. 1d) to isolate AP1^+^ glia (dsRed^+^GFP^+^), AP1^neg^ glia (dsRed^neg^GFP^+^) and neurons (dsRed^neg^GFP^neg^; schematic in Fig. 1h). Overall, dsRed^+^ cells were rare (roughly 1–3% brain cells), nearly all were GFP^+^ (Extended Data Fig. 1e), and dsRed^+^GFP^+^ cells expressed glial marker genes (Extended Data Fig. 1f). Consistent with AP1 activation in AP1^+^ glia, *dFos* and *dJun* (two AP1 subunits) were highest in AP1^+^ glia, as were AP1-target genes (Fig. 1i and Supplementary Data 1). Senescence-associated genes were also notably elevated in AP1^+^ glia (Fig. 1j), consistent with an inflammatory secretory phenotype. Conversely, *LamB*, encoding a nuclear envelope protein decreased in senescent cells^30^, seemed reduced. We explored whether AP1^+^ glia have other traits of senescence: cell cycle arrest, increased metabolic activity, hypertrophy and DNA damage^31^. Adult fly glia have the capacity to divide, evidenced by EdU^+^ glia in the setting of brain injury^15,32^. However, most AP1^+^ glia were EdU^neg^ through late life (40 days; Fig. 1k), indicating they neither arise from dividing cells (replicative senescence) nor give rise to new cells (cell cycle arrest). Pathway enrichment analysis showed many metabolic and anabolic pathway genes are highly expressed in AP1^+^ glia, including glycolysis and translation (see Supplementary Data 2 for all pathways and terms). By flow cytometry analysis, AP1^+^ glia were larger than other cells with normal DNA content (Fig. 1l). AP1^+^ glia had elevated γH2Av staining indicative of DNA damage (Fig. 1m). Collectively, AP1^+^ glia have a phenotype consistent with senescence. Herein, we refer to AP1^+^ glia as senescent glia and, by extension, AP1^neg^ glia as non-senescent glia.

## Neuron health is linked to AP1^+^ glia

**Fig. 2:**
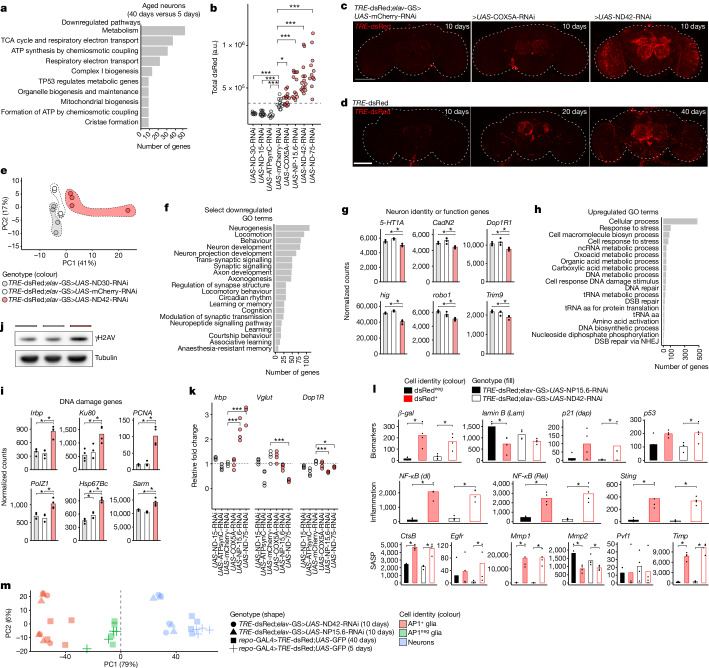
Neuronal mitochondrial dysfunction triggers senescent AP1^+^ glia. **a**, Reactome pathway enrichment shows neuronal mitochondrial function decreases with age. See Supplementary Data 3 and 4 for differential expression genes and gene ontology (GO) terms. **b**, Quantification of dsRed intensity shows knockdown of inner complex genes in neurons increases (red) or decreases AP1 activity (black) relative to control (white) (*TRE*-dsRed;*elav*-GS>*UAS-*RNAi as indicated on the *x* axis; *n* = 11–19 brains per genotype). **c**,**d**, Neuronal knockdown of inner complex genes elicits AP1^+^ glia (**c**) similar to natural ageing (**d**). See Extended Data Fig. 3b,c for quantification and high-magnification images. **e**, PCA of bulk RNA-sequenced brains at 10 days of age (*n* = 20 brains per replicate). **f**–**i**, Neuronal loss of *ND42* reduces neuron-specific processes (**f**) and genes (**g**) with increased DNA damage pathways (**h**) and genes (**i**). See Supplementary Data 6 and 7 for enriched terms and differential expression genes (*TRE*-dsRed;*elav*-GS>*UAS*-ND42-RNAi versus >*UAS*-mCherry-RNAi). **j**, Representative immunoblot showing neuronal *ND42* knockdown increases brain γH2Av levels, legend as in **e**; quantification in Extended Data Fig. 5c. For gel source data, see Supplementary Fig. 1b. **k**, Real-time qPCR of 10-day-old brains shows neuronal loss of *ND75* and *NP15.6* (two AP1-activating RNAi lines, **b**) increases *Irbp* while reducing *Vglut* and *Dop1R* (*TRE*-dsRed;*elav*-GS>*UAS-*RNAi as indicated on the *x* axis; *n* = 20 brains per replicate). **l**, FACS-isolated and bulk RNA-sequenced 10-day-old RNAi-induced dsRed^+^ cells express senescence-associated genes (*n* = 500 cells per replicate). See Supplementary Data 8 for differential expression genes. **m**, PCA shows 10-day-old RNAi-induced dsRed^+^ cells cluster with naturally occurring AP1^+^ glia. For bar graphs (**g**,**i**,**l**), data shown are means. Each point in a microscopy experiment represents one brain, and in immunoblot, real-time PCR and bulk RNA-seq experiments it represents one biological replicate. All data were collected from two or three independent experiments. A two sample *t*-test was used (**b**,**k**). Precise *n* and *P* values are in the Source Data. *P-adjusted <0.05 for sequencing data; ***P < 0.001; **P < 0.01, *P < 0.05 for all other data. All scale bars, 100 μm. Source Data

**Extended Data Fig. 2:**
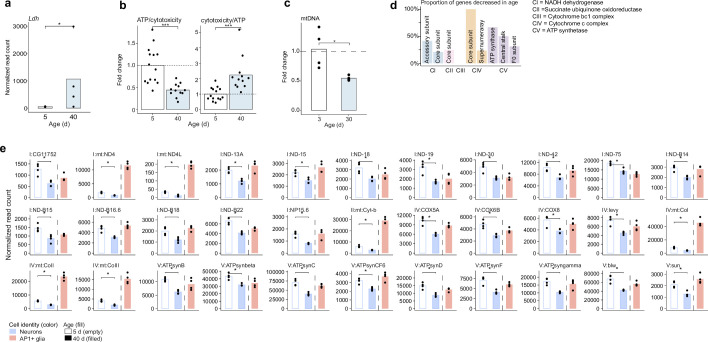
Neuronal mitochondrial function declines in age. **a**, Bulk RNA-sequencing of FACS-isolated neurons shows lactate dehydrogenase expression increases with age (*repo*-GAL4 > *TRE*-dsRed,*UAS*-GFP; *n* = 500 cells per replicate). **b**, Brain ATP, measured as a ratio of ATP to cytotoxicity markers, decreases with age (*w*^*1118*^; *n* = 37 brains). **c**, Total brain mtDNA, measured by PCR, decreases with age (*w*^*1118*^; *n* = 8 brains per replicate). **d**, Schematic showing the complexes associated with the (**e**) 33 inner complex genes reduced in aged neurons, with expression in AP1+ glia shown for comparison. See Supplementary Data 3 for full figure DE genes. For all bar graphs, data are mean. Each point in ATP/cyto toxicity experiment represents one brain, and in mtDNA experiment and bulk RNA-sequencing experiments it depicts one biological replicate. All data were collected from two or three independent experiments. Precise *n* and *P* values are provided in the Source Data. *P-adjusted<0.05 for sequencing data; ***P < 0.001; **P < 0.01, *P < 0.05 for all other data. Source Data

In vitro models have identified potential routes to senescence but why cells senesce in vivo remains unclear^13^. To explore this, we considered the highly stereotyped pattern of AP1^+^ glia: senescent glia first appear in the antennal lobes then the optic lobes. In flies, age-associated loss of smell (antennal lobe neurons) precedes vision loss (optic lobe neurons)^23^, suggesting that glial senescence may coincide with neuronal decline. We FACS-isolated then performed bulk RNA-seq of neurons (dsRed^neg^GFP^neg^) from young brains (5 days) for comparison to aged neurons (40 days). A surprisingly small number of genes changed with age (*n* = 249; Supplementary Data 3). Upregulated genes (*n* = 143) were enriched for inflammation related gene ontology terms, similar to mammalian neurons^33^, whereas downregulated genes (*n* = 106) mostly mapped to pathways and terms related to mitochondrial function (Fig. 2a and Supplementary Data 4). Lactate dehydrogenase messenger RNA increased (Extended Data Fig. 2a), indicating a shift to fermentation. Aged whole brains had reduced ATP levels (Extended Data Fig. 2b), consistent with diminished respiratory gene expression. Total mitochondrial DNA (mtDNA) also decreased (Extended Data Fig. 2c), suggesting reduced mitochondrial content^34^. Overall, these data indicate that mitochondrial function declines with age in neurons, consistent with fly single-cell RNA-seq data^35^ and work in mammalian species and other cell types^33^.

**Extended Data Fig. 3:**
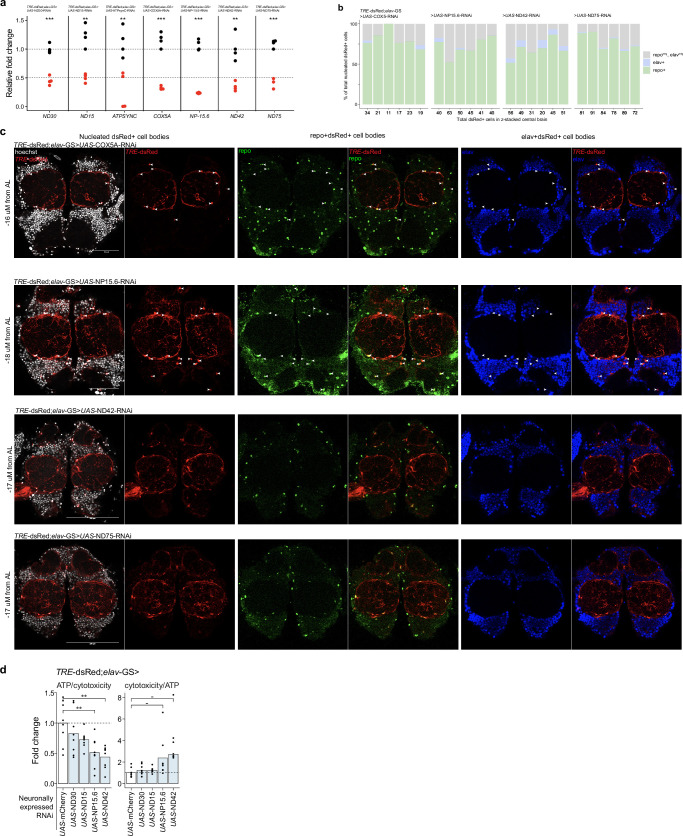
Neuronal knockdown of select inner complex genes activates glial AP1. **a**, Gene expression by real-time qPCR of RNAi target gene levels (x-axis) relative to levels in *TRE*-dsRed;*elav*-GS > *UAS*-mCherry-RNAi control (black) (*n* = 8 brains per replicate). **b**, Proportion of dsRed+ cells in 10 d brains (x-axis shows total number) positive for repo (green), elav (blue) or neither (grey); genotypes as noted in figure. **c**, Representatives high magnification images of 10 d brains showing co-localization of AP1 activity (dsRed) with nuclear glial (repo; middle) and neuronal (elav) antibodies in four of the AP1 activating RNAi lines. **d**, Brain ATP, measured as a ratio of ATP to cytotoxicity markers, in 10 d brains with neuronal RNAi expression relative to *UAS*-mCherry-RNAi. For bar graph (d), data are mean. Each point in ATP/cytotoxicity experiment represents one brain, and in real-time qPCR experiments it depicts one biological replicate. All data were collected from two or three independent experiments. Precise *n* and *P* values are provided in the Source Data. *P-adjusted<0.05 for sequencing data; ***P < 0.001; **P < 0.01, *P < 0.05 for all other data. Source Data

**Extended Data Fig. 4:**
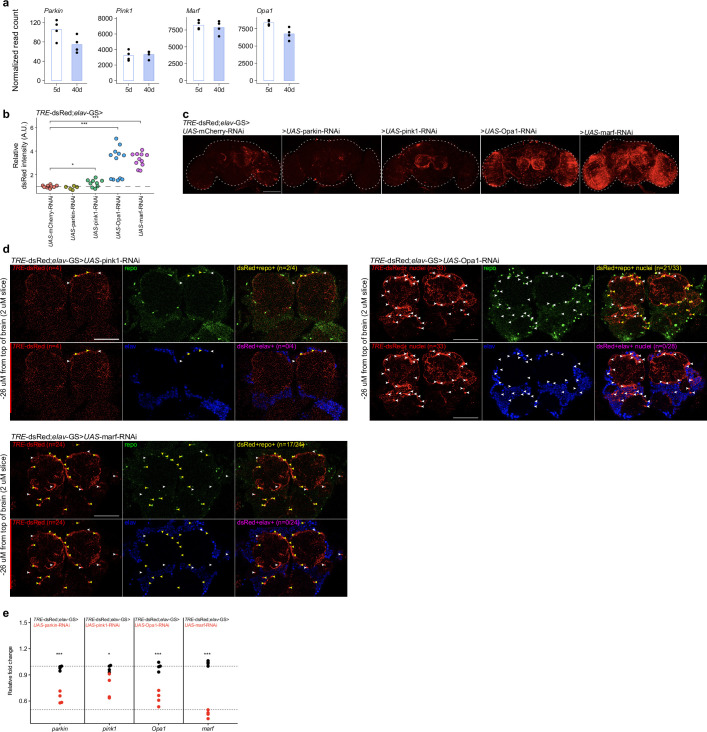
Neuronal knockdown of mitochondrial genes triggers AP1+ glia. **a**, Bulk RNA-sequencing of FACS-isolated neurons shows *parkin, pink1*, *marf* and *Opa1* expression is unchanged with age (*repo*-GAL4 > *TRE*-dsRed,*UAS*-GFP; *n* = 500 cells per replicate). **b**, Quantification of brain dsRed intensity shows that knockdown of mitochondrial genes in neurons increases AP1 activity (black) relative to controls at 10 d age (white) (*n* = 5-11 brains per genotype). Representative images are shown in (**c**) with high-magnification images in (**d**) showing co-localization of AP1 activity with nuclear glial (repo) and neuronal (elav) antibodies. **e**, Quantification of *UAS*-RNAi efficiency by real-time qPCR relative to *UAS*-mCherry-RNAi control (black) (*n* = 8 brains per replicate). For all bar graphs, data are mean. Each point in a microscopy experiment represents one brain, and in real-time qPCR and bulk RNA-sequencing experiments it depicts one biological replicate. All data were collected from two or three independent experiments. Precise *n* and *P* values are provided in the Source Data. *P-adjusted<0.05 for sequencing data; ***P < 0.001; **P < 0.01, *P < 0.05 for all other data. Source Data

In flies, age-onset mitochondrial dysfunction is characterized by inner membrane loss: its complexes and their encoding genes^36^. Of the 46 mitochondrial genes identified by pathway analysis, 33 encoded inner complex components (Extended Data Fig. 2d,e). Thus, to determine whether mitochondrial dysfunction in ageing neurons contributes to glial senescence, we performed a targeted RNA interference (RNAi) screen against the inner complex genes that changed with age in neurons using available RNAi lines (*n* = 20). Experimental or control RNAi were expressed under a drug-inducible (RU-486) neuron-specific GAL4 in a background with the genomic AP1 reporter (*TRE-*dsRed*;elav-*GS). RNAi was induced at adult eclosion and dsRed was assessed at 10 days when AP1 activity is normally low (Fig. 1b). There was an effect in seven lines: three decreased dsRed and four increased dsRed (Fig. 2b); gene knockdown was confirmed in these cases (Extended Data Fig. 3a). In the increased lines, dsRed was mostly glial by repo and elav costaining (Extended Data Fig. 3b,c). Despite driving RNAi in all neurons, when dsRed^+^ glia were seen, they were restricted to the antennal and optic lobes, similar to the pattern seen in normal aged flies: knockdown of *COX5A* and *NP15.6* activated dsRed^+^ glia in the antennal lobes only, as in mid-life (Fig. 2c middle to Fig. 2d middle); *ND42* and *ND75* knockdown phenocopied late life with dsRed^+^ glia also in the optic lobes (Fig. 2c right to Fig. 2d right). Moreover, we found inner complex RNAi lines that increased dsRed compromised brain ATP levels (Extended Data Fig. 3d). We also tested non-inner complex genes vital to mitochondrial health: *pink1*, *parkin*, *marf* and *opa1* (refs. ^37,38^). Expression of these genes was unchanged in aged neurons (Extended Data Fig. 4a). Nonetheless, neuronal knockdown of three out of four genes increased dsRed at 10 days; again, the pattern of dsRed resembled older brains and was glial (Extended Data Fig. 4b–e). Altogether, these data suggest a link between neuronal mitochondrial health and glial AP1 activity.

**Extended Data Fig. 5:**
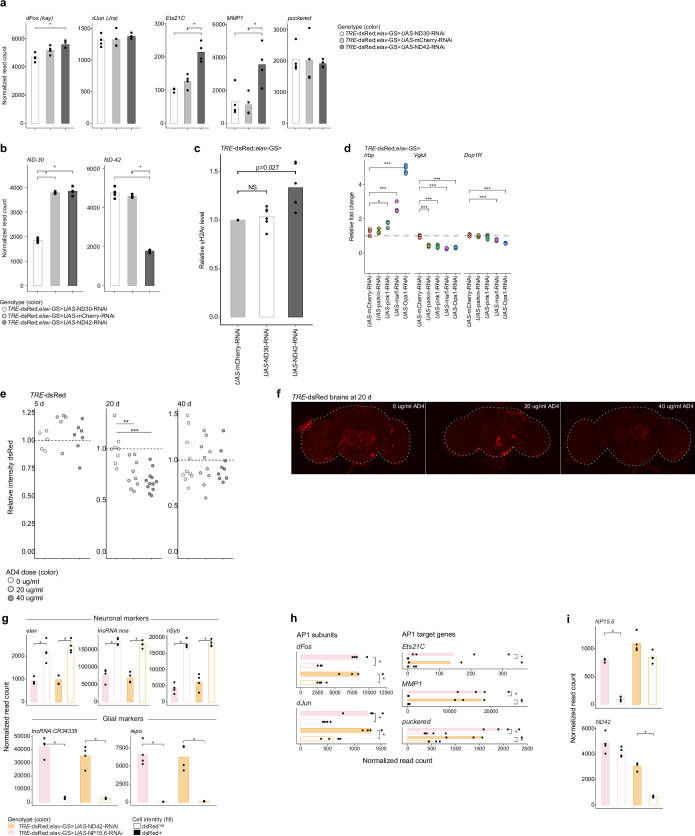
Neuronal mitochondrial health triggers senescent AP1+ glia. **a**, Bulk RNA-sequencing of brains shows that neuronal loss of *ND42* increases AP1 subunits and AP1-target genes (*n* = 20 brains per replicate). Supplementary Data 7 for DE genes. **b**, ND42 and ND30 levels are selectively reduced with neuronal *UAS*-RNAi. **c**, Neuronal loss of *ND42* increases γH2Av protein levels at 10 d age (*n* = 8 brains per replicate). **d**, Real-time qPCR of 10 d brains shows that neuronal knockdown of mitochondrial genes increases *Irbp* while reducing *Vglut* and *Dop1R* (*n* = 20 brains per replicate). **e**, Feeding *TRE*-dsRed flies the anti-oxidant drug AD4 reduces brain AP1 activity by dsRed at 20 d with representative images in (**f**). FACS-isolated and bulk RNA-sequenced 10 ddsRed+ and dsRed^neg^ cells from two AP1-activating inner complex RNAi lines showing (**g**) expression of neuronal and glial marker genes, (**h**) AP1 subunits and AP1 target genes and (**j**) RNAi-targeted genes (*n* = 500 cells per replicate). See Supplementary Data 8 for DE genes. For all bar graphs, data are mean. Each point in a microscopy experiment represents one brain, and in western immunoblot, real-time qPCR and bulk RNA-sequencing experiments it one biological replicate. All data were collected from two or three independent experiments. Precise *n* and *P* values are provided in the Source Data. *P-adjusted<0.05 for sequencing data; ***P < 0.001; **P < 0.01, *P < 0.05 for all other data. Source Data

To gain further insight to these AP1-activating circumstances, we used an unbiased approach and performed bulk RNA-seq on brains from a line with increased dsRed (*TRE*-dsRed;*elav*-GS*>UAS*-ND42-RNAi) and a line with decreased dsRed (>*UAS-*ND30-RNAi). By principal components analysis (PCA), samples segregated along PC1 by dsRed intensity (Fig. 2e). Consistent with dsRed by microscopy, AP1-target genes notably increased with *UAS*-ND42-RNAi (Extended Data Fig. 5a and Supplementary Data 5). RNAi knockdown was specific (Extended Data Fig. 5b). Although we expected *ND42* loss would affect respiration, most downregulated gene ontology terms related to neuron-specific pathways and processes (Fig. 2f,g and Supplementary Data 6), suggesting a loss of cellular identity akin to exdifferentiation^39^. By contrast, upregulated gene ontology terms suggested a DNA damage response (Fig. 2h), with upregulation of canonical repair genes (Fig. 2i). By western immunoblot, *UAS*-ND42-RNAi increased DNA damage associated γH2Av (Fig. 2j and Extended Data Fig. 5c). The line with decreased dsRed, *UAS-*ND30-RNAi, had only one enriched term (eukaryotic translation) and DNA damage and neuronal genes were unchanged (Fig. 2g,i and Supplementary Data 7). These data indicate *ND42* loss caused DNA damage and a loss of neuronal identity, as in normally aged fly and human neurons^33,39^. The mechanism of reduced dsRed by *ND30* loss seems distinct.

To extend these findings, we measured expression of a DNA damage gene, *Irbp*, and neuron-specific genes (*Dop1R*, *Vglut*) in the other nine RNAi lines that affected AP1 activity. In the lines that increased dsRed, *Irbp* increased proportional to dsRed protein; *Dop1R* and *Vglut* decreased (Fig. 2k and Extended Data Fig. 5d). Gene expression was unchanged in lines that decreased or did not affect dsRed. Finally, we fed *TRE-*dsRed flies the antioxidant drug AD4 (*N*-acetylcysteine), which reduces reactive oxygen species (ROS) in the setting of mitochondrial dysfunction^40^. AD4 reduced dsRed at least at 20 days (Extended Data Fig. 5e,f), suggesting this extent of ROS protection attenuates AP1 activity. Collectively, these data indicate that neuronal mitochondrial dysfunction in ageing is a trigger of glial AP1 activity.

To confirm that RNAi-induced AP1^+^ glia express senescent hallmarks, we FACS-isolated and performed bulk RNA-seq on dsRed^+^ and dsRed^neg^ cells from 10-day-old brains from two AP1-activating lines (*TRE*-dsRed;*elav*-GS>*UAS*-ND42-RNAi and >*UAS*-NP15.6-RNAi). RNAi-induced dsRed^+^ cells resembled AP1^+^ glia from aged brains, with high expression of glial markers (Extended Data Fig. 5g), AP1 subunits, AP1-target genes (Extended Data Fig. 5h), senescence-associated genes (Fig. 2l; see Supplementary Data 8 for all differential expression genes) and pathways (Supplementary Data 9). Direct comparison of 10-day-old RNAi-induced dsRed^+^ cells to 40-day-old naturally occurring AP1^+^ glia by PCA indicated highly similar transcriptional profiles, despite vast differences in chronological age (Fig. 2m). RNAi-targeted genes were selectively reduced in dsRed^neg^ cells (Extended Data Fig. 5i), which were mostly neurons based on cell marker genes (Extended Data Fig. 5g). These data indicate that when impaired neuronal mitochondrial function coincides with a signature of neuronal ageing (DNA damage; loss of neural identity), glial AP1 is activated along with a senescence response that resembles natural ageing.

## AP1^+^ glia affect lifespan and health span

**Fig. 3:**
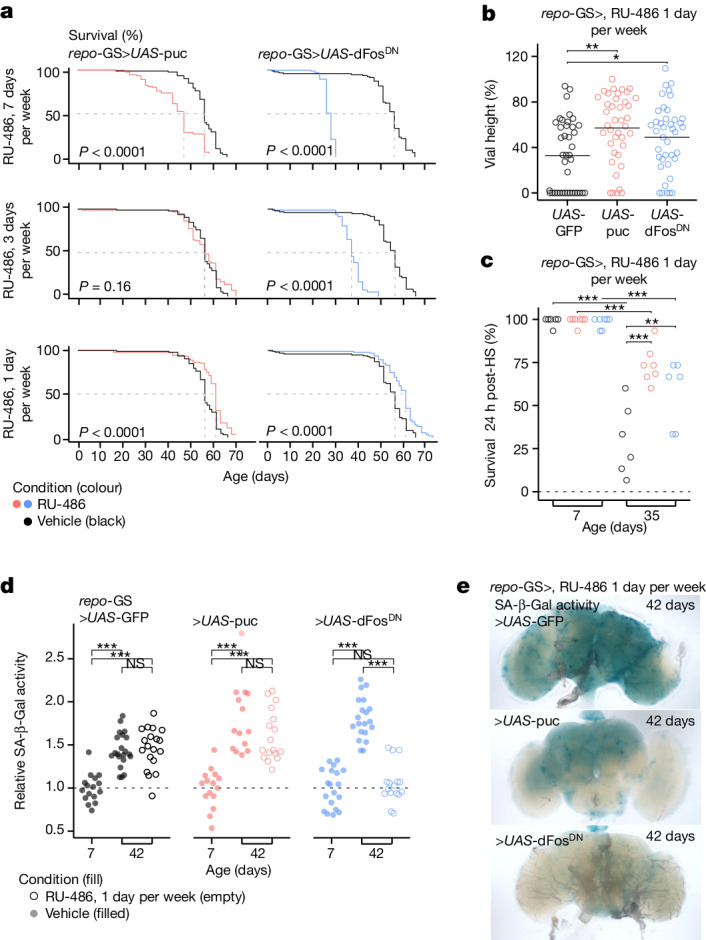
Dampening glial AP1 activity extends animal lifespan and health span. **a**, Lifespan of flies with glial AP1 activity blocked by expressing puckered (*UAS*-puc) or a dominant negative dFos (*UAS*-dFos^DN^) for 7 (top), 3 (middle) or 1 day(s) per week (bottom) (*n* = 100 flies per genotype and condition). Grey lines indicate days animals were fed the geneSwitch activating drug, RU-486. See Extended Data Fig. 6a for comparison to *UAS-*GFP controls. **b**,**c**, Blocking glial AP1 activity for 1 day per week improves climbing ability at 35 days of age, measured by vial height reached after 30 s (*n* = 30) (**b**) and heat shock (HS) survival measured at 24 h after mild heat exposure (*n* = 15 per cohort) (**c**). Legend as in **a**. **d**, Blocking glial AP1 activity for 1 day per week reduces SA-β-Gal activity (*n* = 13–29 brains). **e**, Representative images of **d**. See Extended Data Fig. 6b–e for additional images and experimental conditions. Each point in a microscopy experiment represents one brain, and in climbing experiments it depicts one fly (averaged from three independent measurements), and in heat shock it depicts one cohort of flies. All data were collected from two or three independent experiments. Kaplan–Meier survival with pairwise comparison by log-rank test was used in **a**. Two-way analysis of variance (ANOVA) with Tukey’s comparison was used in **b**,**c**. One-way ANOVA with Tukey’s comparison was used in **d**. Precise *n* and *P* values are provided in the Source Data. *P-adjusted <0.05 for sequencing data; ***P < 0.001; **P < 0.01, *P < 0.05 for all other data. NS, not significant. Source Data

**Extended Data Fig. 6:**
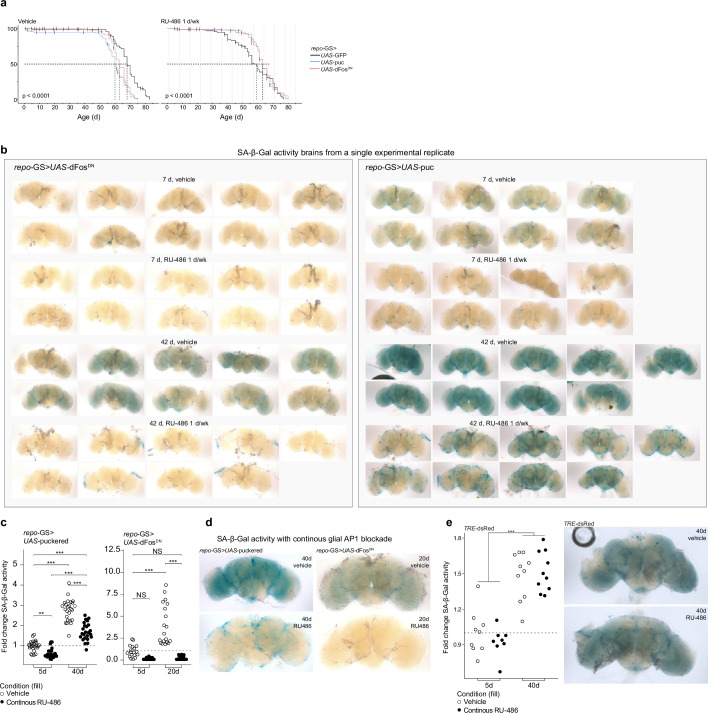
Blocking glial AP1 activity extends lifespan and mitigates SA-β-Gal activity in age. **a**, Lifespan of flies with glial AP1 activity blocked by *UAS-*puc or *UAS-*dFosDN for 1 d/wk relative to *UAS-*GFP controls (*n* = 100 flies per genotype and condition). Grey lines indicate days animals were fed the *geneSwitch* activating drug, RU-486. **b**, Representative images from one experimental replicate showing SA-β-Gal activity decreases when glial AP1 activity is blocked for 1 d/wk (RU-486). **c**, Quantification of brain SA-β-Gal activity with glial AP1 activity blocked for 7 d/wk by expression of puc (left) or dFosDN (right), with representative images shown in (**d**). **e**, Quantification of brain SA-β-Gal activity from flies (*TRE-*dsRed) maintained on vehicle or RU-486 7d/wk from eclosion with representative images (right). Each point in a microscopy experiment represents one brain. All data were collected from two or three independent experiments. Kaplan-Meier Survival with pairwise comparison by Log-Rank test (a). Two-way ANOVA with Tukey’s comparison (c,e). One-way ANOVA with Tukey’s comparison (d). Precise *n* and *P* values are provided in the Source Data. *P-adjusted<0.05 for sequencing data; ***P < 0.001; **P < 0.01, *P < 0.05 for all other data. Source Data

In mice, genetic approaches that continuously eliminate senescent cells impede wound healing, but intermittent elimination (twice a week) extends health span and lifespan^9,17^. We used a similar approach to determine the effect of senescent glia on ageing *Drosophila*. Glial AP1 activity was blocked continuously (7 days per week) or intermittently (3 or 1 day per week) from eclosion of the adult animal, using an inducible glia-specific GAL4 (*repo-*GS; geneSwitch) to express either a dominant negative dFos (*UAS-*dFos^DN^) or an AP1-inactivating phosphatase (*UAS-*puc). Timed feeding of the drug RU-486 was used to start and stop UAS-transgene expression^41^. Continuous AP1 blockade had no effect in early life but by mid-life (roughly 20 days), concurrent with the first appearance of senescent glia in the antennal lobes, survival noticeably declined (Fig. 3a; top). In the case of *UAS*-dFos^DN^, all animals died by 30 days. Intermittent blockade for 3 days per week mitigated this lethality (Fig. 3a; middle). However, blocking AP1 for 1 day per week not only extended median and maximum lifespan beyond controls (vehicle, Fig. 3a, bottom; *UAS-*GFP, Extended Data Fig. 6a), but also improved locomotor activity (Fig. 3b), heat stress recovery (Fig. 3c) and reduced brain SA-β-Gal activity in late life (Fig. 3d,e and Extended Data Fig. 6b), albeit to a lesser extent by *UAS*-puc than *UAS*-dFos^DN^. Continuous blockade with either construct significantly reduced SA-β-Gal activity (Extended Data Fig. 6c,d); RU-486 alone had no effect (Extended Data Fig. 6e). Overall, these data indicate glial AP1 is essential as the fly ages, but mildly dampening AP1 activity (1 day per week) improves animal lifespan and health span relative to normal ageing, similar to the benefits of targeting senescent cells in mice.

## AP1^+^ glia promote lipid accumulation

**Fig. 4:**
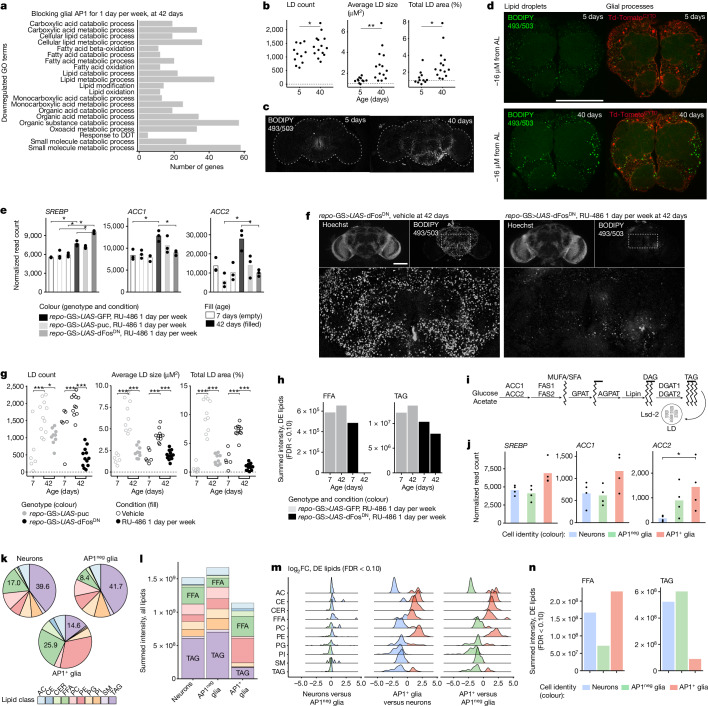
AP1 contributes to FFAs in AP1^+^ glia and TAGs in AP1^neg^ glia. **a**, Bulk RNA-seq of brains shows that blocking glial AP1 activity for 1 day per week reduces lipid metabolism genes at 42 days of age (analysis on shared differential expression (DE) genes between repo-GS>*UAS-*dFos^DN^ versus >*UAS*-GFP and *repo*-GS > *UAS-*puc versus >*UAS*-GFP). See Supplementary Data 9 and 10 for terms and differentially expressed genes. **b**,**c**, BODIPY^+^ LDs increase with age (*TRE*-dsRed; *n* = 13–15 brains) (**b**) with representative images (**c**). **d**, Representative images showing most BODIPY^+^ LD (green) are in glia (red; *repo*-GAL4>*UAS*-tdTomato^CYTO^). See Supplementary Video 1 and Extended Data Fig. 7f–i for additional experiments. AL, antenna lobes. **e**–**g**, Blocking glial AP1 activity for 1 day per week reduces lipogenesis genes (**e**) and BODIPY^+^ LD with age (**f**), with quantification (**g**) (*n* = 55 brains). See Extended Data Fig. 8a,b for additional genes and images. **h**, Lipidomic analysis shows that blocking glial AP1 activity for 1 day per week reduces brain FFA and TAG abundance (*n* = 8 brains per replicate). See Extended Data Fig. 8c–e for additional data. **i**, Schematic of the de novo lipogenesis pathway; proteins in black and lipids in grey text. **j**, AP1^+^ glia express lipogenesis genes (cells as in Fig. 1i). **k**,**l**, Lipidomic analysis of FACS-isolated cells from 40-day-old brains (as in Fig. 1h) shows that AP1^+^ glia have a distinct composition by relative (**k**) and total lipid abundance (**l**). **m**,**n**, Analysis of differentially expressed lipids by log fold change (**m**) and summed intensity (**n**) shows that AP1^+^ glia have more FFA but fewer TAGs than AP1^neg^ glia (*n* = 100,000 neurons, *n* = 100,000 AP1^neg^ glia, *n* = 35,000 AP1^+^ glia per replicate). Bar graphs in **e**,**j** represent mean and in **h**,**l**,**n** represent summed values across 5–6 biological replicates. Each point in a microscopy experiment represents one brain, and in bulk RNA-seq experiments it depicts one biological replicate. Lipid class key: AC, acyl carnitine; CE, cholesteryl ester; CER, ceramide; PC, phosphatidylcholine; SM, sphingomyelin; PE, phosphatidylethanolamine; PG, phosphatidylglycerol; PI, phosphatidylinositol. Precise *n* and *P* values are provided in the Source Data. *P-adjusted <0.05 for sequencing data; false discovery rate (FDR) <0.10 for lipidomic data; ***P < 0.001; **P < 0.01, *P < 0.05 for all other data. Source Data

**Extended Data Fig. 7:**
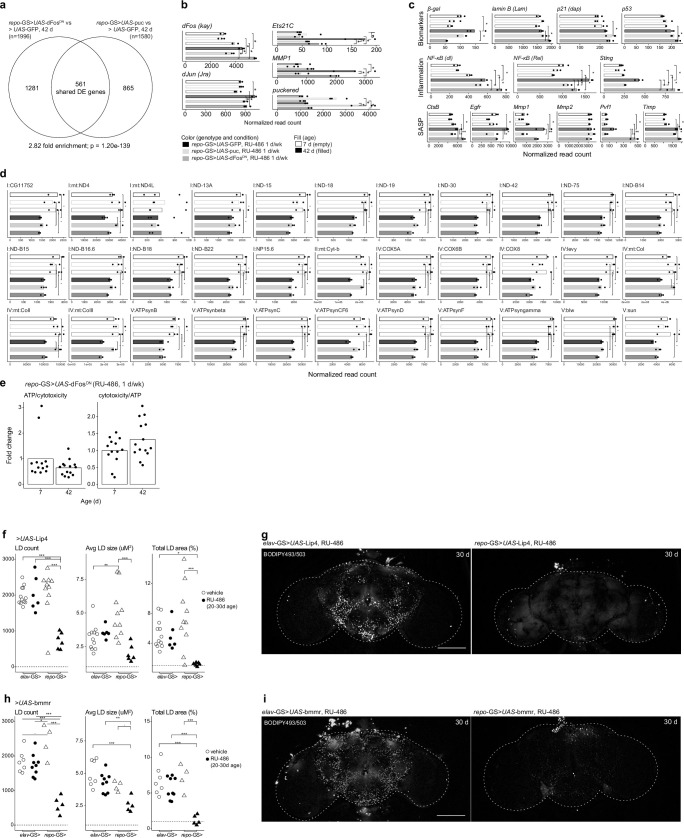
Blocking glial AP1 for 1 d/wk reduces senescence biomarkers in the aging brain, but does not improve mitochondrial health. **a**, Hypergeometric comparison showing there is a significant overlap of DE genes between the two AP1 targeting complexes, *UAS-*puc or *UAS-*dFosDN. **b**, Blocking glial AP1 activity reduces age-onset expression of (**b**) AP1 subunits, AP1-target genes and (**c**) senescence biomarkers in age while (**d**) inner complex genes are unchanged. See Supplementary Data 10 for DE genes. **e**, Age-onset reduction in brain ATP, measured as a ratio of ATP to cytotoxicity markers, persists age in the setting of blocking glial AP1 activity (*n* = 27 brains). TAG lipase (**f**, Lip4 or **h**, bmmr) expression starting from age 20 d reduces BODIPY + LD at 30 d when expressed in glia (*repo*-GS >) but not neurons (*elav*-GS >), with representative images shown in (**g**,**i**). For all bar graphs, data are mean. Each point in a microscopy and ATP/cyto toxicity experiments represents one brain, and in bulk RNA-sequencing experiments it depicts one biological replicate. All data were collected from two or three independent experiments. Precise *n* and *P* values are provided in the Source Data. *P-adjusted<0.05 for sequencing data; ***P < 0.001; **P < 0.01, *P < 0.05 for all other data. Source Data

To understand the biologic and molecular effect on the brain when we target senescent glia and extend animal health, we bulk RNA-sequenced the brains of young (7 days) and aged (42 days) flies, with or without intermittent glial AP1 blockade (*repo-*GS>*UAS-*dFos^DN^ or >*UAS*-puc versus >*UAS-*GFP; RU-486 1 day per week). We saw a significant overlap of differentially expressed genes between AP1 targeting constructs, suggesting a similar effect (Extended Data Fig. 7a; Supplementary Data 10 lists shared differential expression genes). Consistent with reduced AP1 activity, AP1-target genes decreased (Extended Data Fig. 7b), as did senescence biomarkers (Extended Data Fig. 7c). However, markers of mitochondrial status—inner complex gene expression and brain ATP levels—were unchanged (Extended Data Fig. 7d,e). Thus, targeting glial AP1 did not alter neuronal mitochondrial decline with age; these data are consistent with glial senescence being a secondary response. Rather, the most notable change was a downregulation of lipid metabolism-related genes and processes (Fig. 4a and Supplementary Data 11), including proteins that make and store free fatty acids (FFAs) as triacylglycerides (TAGs) in LDs.

**Extended Data Fig. 8:**
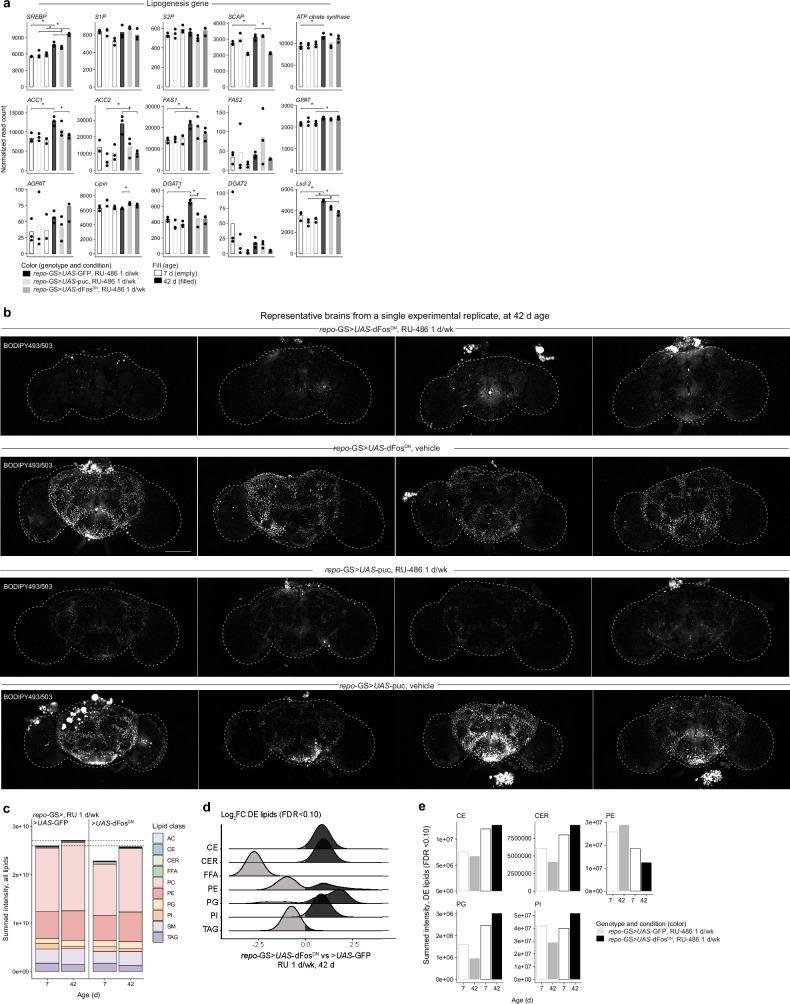
Intermittent glial AP1 blockade reduces lipogenesis and lipid accumulation with age. **a**, Blocking glial AP1 activity for 1 d/wk reduces age-onset increase of lipogenesis and LD biosynthesis genes by bulk RNA-sequencing of whole brains (*n* = 20 brains per replicate). **b**, Representative images from one experimental replicate showing that blocking glial AP1 activity for 1 d/wk reduces age-onset increase in BODIPY + LD. **c**, Total intensity of all measured lipids in brains according to class. Analysis of differentially expressed lipids by (**m**) log fold-change and (**n**) summed intensity shows that blocking glial AP1 for 1 d/wk reduces FFA and TAG at 42 d age (*n* = 8 brains per replicate). Bar graphs in (a) represent mean; bars graphs in (c,e) represent summed values across 5-6 replicates. Each point in a microscopy experiment represents one brain, and in bulk RNA-sequencing experiments it depicts one biological replicate. See methods for lipid key. Precise *n* and *P* values are provided in the Source Data. *P-adjusted<0.05 for sequencing data; FDR < 0.10 for lipidomic data; ***P < 0.001; **P < 0.01, *P < 0.05 for all other data. Source Data

LDs have been increasingly implicated in mammalian brain ageing and disease; they accumulate with age^42^, LD-laden glia promote pathology^43^ and variants in lipid-related genes confer disease risk^44^. We extended these findings to the fly brain by performing BODIPY staining for neutral lipids (TAGs) in normal ageing brains. BODIPY^+^ LD were present in young brains, mostly in the central brain, and increased in size and number with age (Fig. 4b,c). To identify the cells in which LDs accumulate, we depleted TAGs in neurons or in glia by expressing a TAG lipase from age 20 to 30 days: glial lipase notably reduced LDs whereas neuronal lipase had no effect (Extended Data Fig. 7f–i), indicating TAGs accumulate in glia with age. Microscopy confirmed that BODIPY^+^ LD were enriched along tdTom^+^ glial processes and absent from neuron-rich neuropil (Fig. 4d and Supplementary Video 1). Although lipogenesis and LD biosynthesis genes increased with normal brain ageing, coinciding with LD accumulation, gene expression was reduced in the setting of AP1 blockade (Fig. 4e and Extended Data Fig. 8a). When we performed BODIPY staining, we found that intermittent AP1 blockade also notably reduced BODIPY^+^ LDs at 42 days of age (Fig. 4f,g and Extended Data Fig. 8b), to an extent comparable to glial lipase expression. Whole-brain lipidomic profiling confirmed that TAGs decreased, consistent with LD loss. There was also a striking reduction in FFAs (Fig. 4h and Extended Data Fig. 8c–e), suggesting LD reduction in the setting of AP1 blockade may be secondary to reduced lipid synthesis.

**Extended Data Fig. 9:**
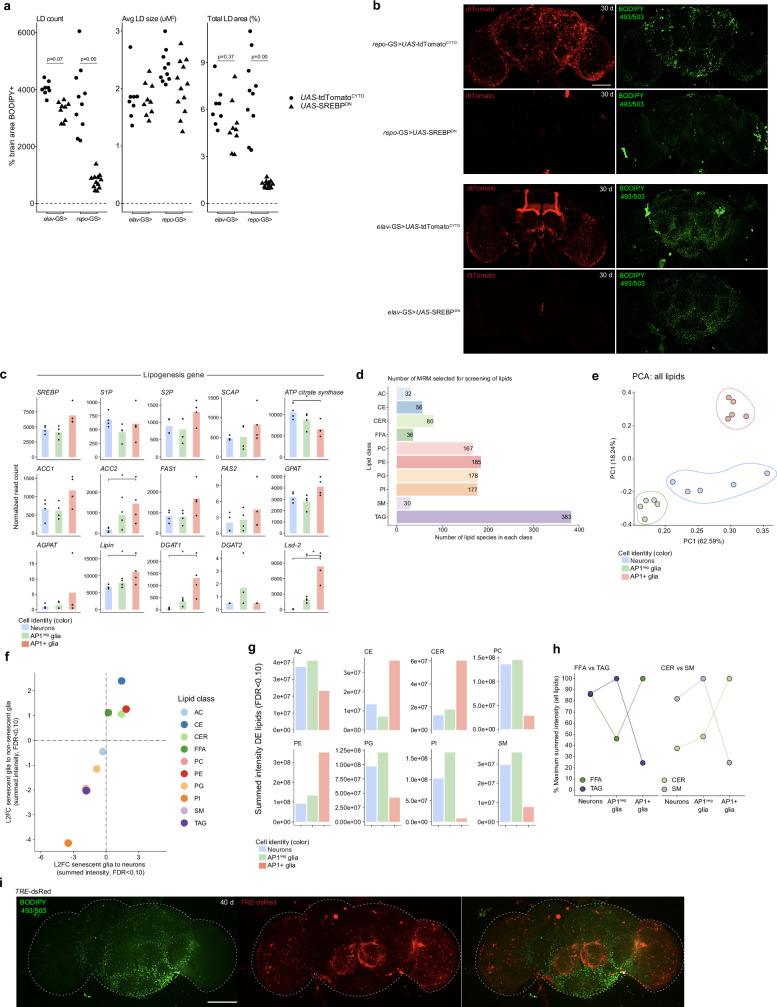
FFAs are abundant in AP1+ glia while TAGs are abundant in AP1^neg^ glia. **a**, Blocking SREBP activity by SREBP^DN^ expression from age 20 d reduces BODIPY + LD at 30 d when expressed in glia (*repo*-GS >) but not neurons (*elav*-GS >), with representative images shown in (**b**). **c**, Lipogenesis pathway genes (FFA to lipid droplet) in bulk RNA-sequenced FACS-isolated cells from aged brains (data as in Fig. 1h–j). **d**, Total number of lipid species screened in FACS-isolated cells and whole brains, according to lipid class. **e**, Principal component analysis of lipidomic profiling of FACS-isolated cells, where each point represents one biological replicate (*n* = 100,000 neurons, *n* = 100,000 AP1^neg^ glia, *n* = 35,000 AP1+ glia per replicate). **f**, Log_2_ fold change of summed intensity of DE lipids by class between FACS-isolated cell populations with the comparison as indicated on y or x axis. **g**, Summed intensity of DE lipids by lipid class, with differential comparison defined as FDR < 0.10 for AP1+ glia vs neurons or AP1+ glia vs AP1^neg^ glia. **h**, Maximum summed intensity, where maximum value was defined by the highest summed intensity among three cell populations for a given lipid class. **i**, Representative image showing BODIPY + LD do not co-localize with AP1+ glia by dsRed. Bar graphs in (c) represent mean; data in (g-h) represent summed values across biological replicates. Each point in a microscopy experiment represents one brain, and in bulk RNA-sequencing experiments it depicts one biological replicate. Precise *n* and *P* values are provided in the Source Data. *P-adjusted<0.05 for sequencing data; FDR < 0.10 for lipidomic data; ***P < 0.001; **P < 0.01, *P < 0.05 for all other data. Source Data

In the fly retina, pigment cell LDs accumulate lipids synthesized by neurons^40^. To identify the source of lipids that accumulate in glial LDs with age, we blocked the master lipogenesis transcription factor, SREBP, in neurons or glia starting from 20 days of age. Glial, but not neuronal, SREBP perturbation eliminated LDs at 30 days (Extended Data Fig. 9a,b), indicating glial lipogenesis contributes to glial LDs. When we examined lipogenesis genes in FACS-isolated aged cell populations, de novo lipogenesis genes (FFA/TAG; diagram in Fig. 4i) were highest in AP1^+^ glia (Fig. 4j and Extended Data Fig. 9c), identifying senescent fly glia as lipogenic, consistent with in vitro characterization^45,46^. To further investigate lipid content, we performed lipidomic profiling on FACS-isolated cells from 40-day-old brains (neurons, AP1^neg^ glia, AP1^+^ glia; Extended Data Fig. 9d). Overall, neurons and AP1^neg^ glia had a similar lipid composition whereas AP1^+^ glia were distinct (Fig. 4k–m and PCA in Extended Data Fig. 9e). AP1^+^ glia had the highest levels of most FFA species (34 of 35 measured species), amounting to roughly twofold greater abundance (Fig. 4n); these data suggest senescent glia may be a source of excess FFAs. Yet, TAGs and sphingomyelins (LD lipids) were highest in AP1^neg^ glia, then neurons, but were unexpectedly low in senescent AP1^+^ glia (Fig. 4n and Extended Data Fig. 9e–h), suggesting LDs are in AP1^neg^ glia. Indeed, microscopy showed BODIPY^+^ LDs rarely colocalized with AP1 activity by dsRed (Extended Data Fig. 9i and Supplementary Video 2). Altogether, these data show FFAs are replete in AP1^+^ glia whereas TAGs accumulate in AP1^neg^ glia. Targeting glial AP1 affects AP1^+^ and AP1^neg^ glia, reducing FFAs and TAGs or LDs, respectively.

**Fig. 5:**
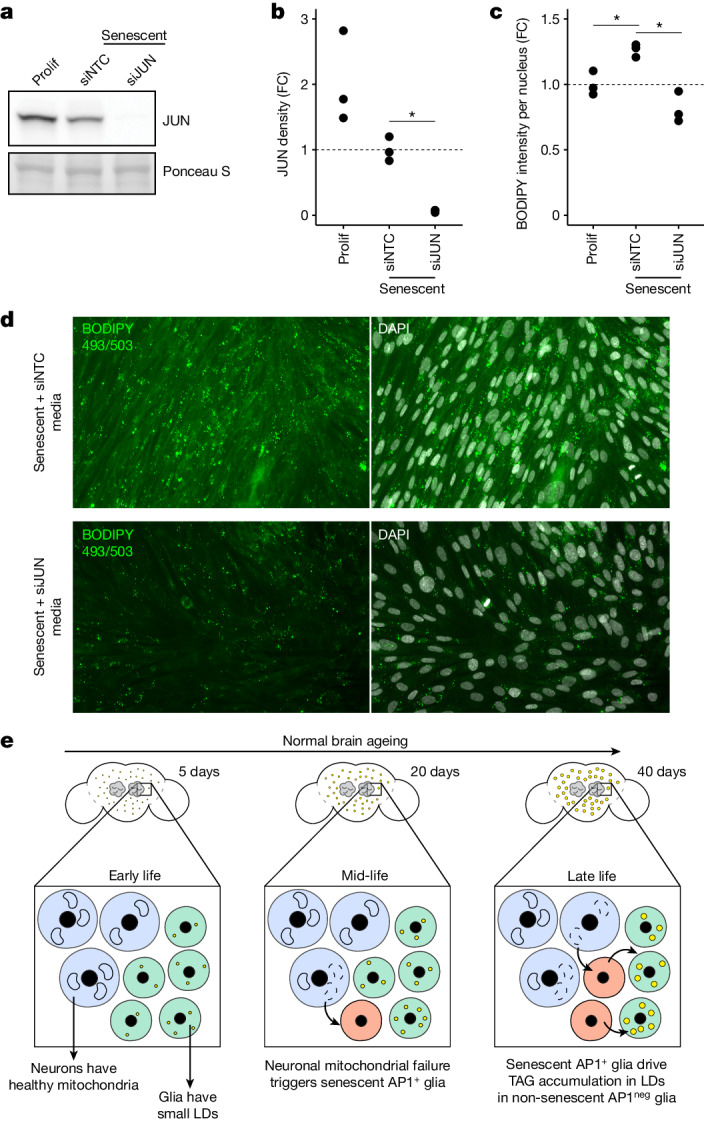
JUN in senescent human cells promotes LDs in non-senescent cells. **a**,**b**, Representative western immunoblot (**a**) and quantification (**b**) of JUN protein in proliferating IMR90 cells (left) compared to senescent IMR90 cells treated with siJUN or siNTC (non-targeting control). For gel source data, see Supplementary Fig. 1c. **c**,**d**, Quantification of BODIPY 493/503 intensity in proliferating IMR90 cells (**c**) treated with conditioned medium from indicated conditions with representative images (**d**). **e**, Model for interaction between neurons (blue), AP1^neg^ glia (green) and AP1^+^ glia (red); declining mitochondrial function in neurons triggers AP1 activity in AP1^+^ glia with age, which promotes LD (yellow) accumulation in AP1^neg^ glia. Each point represents one biological replicate from three independent experiments. One-way ANOVA with Tukey’s comparison (**b**,**c**). Precise *n* and *P* values are provided in the Source Data. *P-adjusted <0.05 for sequencing data; ***P < 0.001; **P < 0.01, *P < 0.05 for all other data. FC, fold change. Source Data

These data suggested that AP1 activity in senescent cells can affect lipid accumulation in non-senescent cells. To further address this, we turned to mammalian cell culture. IMR90 cells were irradiated to induce senescence, then transfected with either *JUN-*targeting short interfering RNA (siJUN) (to knockdown AP1) or a non-targeting control (siNTC). Knockdown efficiency of JUN protein was more than 90% (Fig. 5a,b). Cell culture medium was collected 10 days after irradiation and transferred to naive IMR90 cells for 48 hours. Medium from siNTC transfected cells induced LD accumulation by BODIPY staining whereas medium from siJUN-treated cells had reduced LDs. These data indicate LD induction is non-cell autonomous and promoted by AP1 (Fig. 5c,d). Taken together with the data from the fly, these findings suggest a model in which senescent glia produce factors dependent on AP1 that promote LDs in non-senescent glia (Fig. 5e), reshaping cell and tissue biology with age.

**Extended Data Fig. 10:**
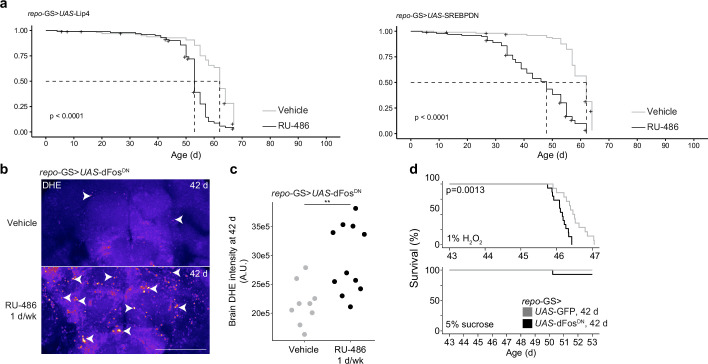
Blocking glial AP1 activity for 1 d/wk increases oxidative markers and reduces H2O2 resilience. **a**, Expressing Lip4 (left) or SREBP^DN^ (right) in glia for 7 d/wk reduces animal lifespan (*n* = 100 flies per condition). **b**, Brain DHE staining at 42 d increases when glial AP1 blocked for 1 d/wk (RU-486), with quantification in (**c**). **d**, Blocking glial AP1 activity for 1 d/wk reduces animal resilience to 1% H2O2 feeding. Data represent mean survival (*n* = 20, two independent experiments). Each point in a microscopy experiment represents one brain. Precise *n* and *P* values are provided in the Source Data. *P-adjusted<0.05 for sequencing data; ***P < 0.001; **P < 0.01, *P < 0.05 for all other data. Source Data

To determine whether LD reduction when targeting glial AP1 is beneficial, we examined additional features of the brain and animals. Glial LDs protect the brain from damage by peroxidated lipids in the setting of mitochondrial ROS^47^. Consistent with a benefit, eliminating LDs by SREBP^DN^ or lipase expression was deleterious (Extended Data Fig. 10a). To investigate the effect of LD loss by AP1 mitigation on oxidative resilience, we stained for DHE, a marker of oxidative damage, and challenged flies with an oxidative stressor (H_2_O_2_ feeding). Despite extended organismal lifespan, the number of DHE^+^ cells in 42-day-old brains increased notably (Extended Data Fig. 10b,c), and AP1 blocked animals were more susceptible to oxidative stress (H_2_O_2_ feeding; Extended Data Fig. 10d). In sum, these data show targeting senescent glia alters lipid biology of the ageing brain, reducing lipid synthesis and LDs, which has both positive (extended animal lifespan and health span) and negative (greater oxidative damage) effects.

## Discussion

We provide an in vivo characterization of naturally occurring senescent glia in *Drosophila*, identifying one cause of senescent cells and their effect on ageing tissue: promoting lipid accumulation. As in mammals, senescent glia in flies appear with age in a stereotyped and regionalized manner^24^, show biomarkers and traits of senescence^13^ and are marked by AP1 activity^14^. Using RNA-seq and a targeted RNAi screen, we determine that loss of select mitochondrial genes in neurons can trigger the senescence of glia. This neural gene loss coincides with greater DNA damage and a loss of neuronal identity, resembling the natural ageing process of human neurons^33^. How neuron health drives glial senescence remains to be determined: aged neurons could release ROS, mtDNA^48–50^ or extrude damaged mitochondria^49–51^, all of which can induce senescence in vitro^21,52–54^. Whether attenuating neuronal demise can delay glial senescence remains to be determined, as bolstering mitochondrial health in ageing neurons has proven elusive^34,55^, despite the ability to boost function in disease^40,56^. These findings represent one path to senescence in vivo; others probably exist. Targeting glial AP1 activity had a dose-dependent effect on animal health, whereby complete blockade of AP1 was deleterious, but mild dampening extended lifespan and health span, consistent with intermittent senolysis in mice^8^. We determined that mildly dampening glial AP1 activity also reduces LD accumulation in non-senescent glia. This finding extended to mammalian cells in culture. These data suggest that senescent cells in vivo may alter lipid storage in non-senescent cells with implications for age-onset disease in mammalian species and in other tissues.

Among the intriguing questions raised by these data is how senescent glia promote LDs in other glia. Senescent glia have a signature of increased lipogenesis, similar to mammalian senescent cells^46^, and are enriched in FFAs; TAGs and LDs are enriched in non-senescent glia. Although TAGs could be used by senescent glia^45^, resulting in low TAG or LD content, it is also possible that FFAs or downstream lipid species ultimately accumulate in other cells. Such cell-to-cell lipid transfer could occur by diffusion^57^, autophagic efflux^58^ or lipid-binding apolipoproteins^59^. Alternatively, senescent glia may produce factors that promote LDs indirectly. Whereas targeting senescent glia is beneficial to animals and eliminates LDs, the effect of LDs in brain ageing is less clear. Our data and others indicate LD may be beneficial: indeed, LDs protect cells from ROS by sequestering harmful peroxidated lipids^47^. However, excess glial LDs may impede phagocytosis^60^ and promote tau aggregation^43^. Overall, although targeting senescent glia has an organismal benefit, it fails to address a core issue in ageing–mitochondrial dysfunction in neurons. Effective anti-ageing strategies may also require bolstering mitochondrial function in neurons.

## Methods

### Fly stocks and maintenance

Flies were raised at 25 °C and 60% relative humidity on standard cornmeal fly food under a 12–12 h light–dark cycle. All experiments were performed with male flies to minimize biological variance, as male and female flies age at notably different rates. Flies were transferred to fresh food vials every 48 h, housed in cohorts of 20 and randomly assigned to experimental conditions. For geneSwitch (inducible GAL4-*UAS*)^41^ experiments, food was prepared with either 100 μl of RU-486 (4 mg ml^−1^ in 100% EtOH; Sigma-Aldrich, M8046-1G) or vehicle (100% EtOH), pipetted onto food vials and allowed to dry for 24 h. See Supplementary Table 1 in the Supplementary Information for genotypes and stock information.

For neuronally expressed *UAS*-RNAi experiments, flies were collected onto RU-486 food at adult eclosion (0 days of age) and reared at 29 °C.

For AP1 blockade experiments, flies were collected onto vehicle or RU-486 food at adult eclosion (0 days of age). Animals were maintained on RU-486 continuously (7 days per week) or intermittently (3 or 1 day per week) by flipping flies to vehicle food. All experiments and sample collection for 1 day per week RU-486 treated flies were performed with animals on vehicle food, specifically at 6 days after last RU-486 feeding to ensure geneSwitch termination^41^. Controls were selected on the basis of assay. For BODIPY experiments, genotype-matched vehicle-fed animals were used as controls, as GFP interferes with BODIPY detection.

### Behavioural assays

To measure survival, the number of dead and/or censored flies was recorded every 2 days after flipping flies to fresh food; flies were housed in vials of 20 each, with a minimum of 100 flies per genotype and experiment, repeated a minimum of two times. To measure climbing, flies were single-housed in empty vials and allowed to acclimate for 30 min. Climbing was measured by tapping flies to the bottom of the vial then recording height climbed after 30 s over three trials with a 5 min testing interval. Averaged climbing height was determined in Fiji. Data are expressed as a percentage of the maximum vial height (8 cm). Heat shock assessment was performed as described in ref. ^61^. In brief, flies were transferred to clear plastic 13 ml vials, and each vial contained 15 flies. Vials plus flies were transferred to a water bath for 1 h at 38.5 °C for stress. The flies were then transferred to fresh food and allowed to recover overnight at 25 °C then the percentage of flies alive versus dead were recorded per vial. Oxidative stress (H_2_O_2_ feeding): adult flies were single-housed and loaded in the *Drosophila* Activity Monitoring system on either 5% sucrose-agar or 1% H_2_O_2_ sucrose-agar. Activity was recorded for 10 days then analysed in the R environment using the rethomics package^62^ to determine time of animal death and generate survival curves.

### FACS-based isolation of AP1^+^ glia, AP1^neg^ glia and neurons for bulk RNA-seq or lipidomic analysis

All work was performed in RNAse-free conditions. To create a cell suspension for FACS-based sorting, adult fly brains (*n* = 20 brains per replicate for RNA-seq; *n* = 40 brains per replicate for lipidomic analysis) were rapidly dissected in Schneider’s medium with 45 μM actinomycin D and stored on ice until dissections were complete. Brains were then washed in cold phosphate-buffered saline (PBS) (3×). A single cell suspension was achieved by enzymatic and physical dissociation as follows: whole brains were incubated in dissociation buffer (300 μl of activated papain, Worthington PAP2 LK003178 and 4.1 μl liberase, Roche 5401119001) at 25 °C at 1,000 rpm on a shaker for a total of 20 min. During incubation, at 5 and 10 min, tissue was gently homogenized by pipetting. At 15 min, the entire homogenate was passed through a 25G 5/8 needle (7×). At 20 min, enzymatic activity was halted by the addition of cold Schneider’s medium. Cells were then strained (35 μM filter), pelleted (800*g*, 7 min) and resuspended in cold Schneider’s medium with actinomycin D and 2.5 μl of RNAse inhibitor (Takara Recombinant RNase Inhibitor, catalogue no. 2313A). Cells were resuspended in 250 μl, counterstained with 5 μM 4,6-diamidino-2-phenylindole (DAPI) and 50 nM syto60 (nuclear marker; ThermoFisher, catalogue no. S11342) and sorted by the Penn Cytomics and Cell Sorting Facility using a BD FACS Aria II SORP (100 μM nozzle; purity). Dead cells were excluded through DAPI uptake. Doublets were excluded through FSC-H by FSC-W and SSC-H by SSC-W parameters. Nucleated cells were included by syto60. Glia were identified by GFP, and neurons were GFP negative. AP1 activity was identified by dsRed. The gating strategy is shown in Extended Data Fig. 1c.

For bulk RNA-seq, 500 neurons, 500 AP1^+^ glia and 500 AP1^neg^ glia were collected per replicate, with four replicates per cell type. For lipidomic profiling, 100,000 neurons, 100,000 AP1^neg^ glia and 35,000 AP1^+^ glia were collected per replicate, with 5–6 replicates per cell type. Cells were immediately frozen at −80 °C until further processing. Total processing of tissue and cell isolation took roughly 3 h. Data from the sort were analysed using FlowJo v.10.8.1. To generate cell and DNA content plots, cell populations with different *N* were overlaid using absolute cell counts normalized to mode (to the peak height at mode of the distribution).

For immunostained FACS-isolated cells, following dissociation and resuspension as above, cells were fixed in 4% paraformaldehyde for 15 min at room temperature. Cells were washed then resuspended in 5% normal goat serum (NGS) for 5 min on ice. Cells were next incubated in primary antibody (1:20 mouse anti-γH2Av, DSHB UNC93-5.2.1) for 30 min at room temperature, washed and incubated in secondary antibody (1:200 goat antimouse AlexaFluor 647, ThermoFisher Scientific, catalogue no. A-21235) for 30 min at room temperature. Gating parameters were as above. Cells were immediately analysed using a BD FACS Aria II SORP as above.

### Bulk RNA-seq and analysis

For sorted cells, RNA isolation, library preparation (SMART-Seq v.4) and RNA-seq (Illumina 2 × 150 40 million paired-end reads per sample; 20 million each direction) were performed by Admera Health. For whole brains, roughly 10–12 brains were dissected per condition. Total RNA was extracted using the Zymo RNA clean & concentrator−5 kit (Zymo, R1013), using their RNA clean-up from the aqueous phase after Trizol/chloroform extraction protocol plus on-column DNaseI treatment. RNA amount was measured by nanodrop, and integrity was validated by an Agilent 2100 Bioanalyzer using an RNA nano chip. The RNA-seq libraries (TruSeq stranded with Poly-A selection) and sequencing (Illumina NovaSeq S4 with 40 million paired-end reads; 2 × 150 bp) were performed by Admera Health. Four biological replicates were generated for each sample type, experimental timepoint, condition and genotype.

Demultiplexed reads passing the quality control filter (*Q* > 30) were obtained from BaseSpace then merged across sequencing lanes for each sample, with roughly 20 million reads total per sample. Paired-end reads were aligned to the fly genome using HISAT2 (v.2.1.0)^63^. The HISAT2 index was built from FlyBase’s *Drosophila melanogaster* reference genome r6.17. Alignment sorted BAM files (samtools v.15) for each sample were merged across sequencing runs (picard)^64^. Reads that uniquely aligned to exonic regions were counted with HTSeq (v.0.9.1)^65^ with the union setting to produce a count matrix for differential expression analysis using the DESeq2 (ref. ^66^) package in the R environment. The design model formula was ‘~group’ if there were two or more key variables involved (that is, genotype and age) or design model formula was the single key variable (that is, genotype). Pairwise comparisons were made between samples (that is, ‘contrast=c(’group’)’), with an alpha cut-off of 0.05 with lfcShrink() applied. Gene ontology and Reactome pathway enrichment were performed with tools at Flymine.org, using all expressed genes as background (*n* roughly 15,694). Refer to indicated Supplementary Data for differentially expressed genes between samples and/or groups across experiments.

### Unbiased lipidomics for multiple reaction monitoring profiling and analysis

For lipidomic profiling cells were FACS-isolated as above. Brains were rapidly dissected in PBS, pelleted by centrifugation and excess PBS was removed for freezing at −80 °C until further processing (*n* = 8 brains per replicate; 5–6 replicates per genotype and/or age). Lipid extracts from FACS-sorted cells and whole-brain samples were prepared using a slightly modified Bligh & Dyer extraction procedure^67^. In brief, the frozen samples were thawed for 10 min at room temperature, and 200 μl of ultrapure water was added to promote lysis, followed by 450 μl of methanol and 250 μl of high-performance liquid chromatography-grade chloroform. Samples were vortexed for 10 s, resulting in a one-phase solution, and incubated at 4 °C for 15 min. Next, 250 μl of ultrapure water and 250 μl of chloroform were added, creating a biphasic solution. The samples were centrifuged at 14,000*g* for 10 min, which resulted in three phases in the tubes. The bottom organic phase containing the lipids was transferred to new tubes, then evaporated in a vacuum concentrator leaving behind the dried lipid extracts.

Multiple reaction monitoring profiling of the extracted lipids was performed as described previously^68^. The dried lipid extracts were dissolved in 100 μl of methanol:chloroform (3:1 v/v) to make lipid stock solutions. The lipids were further diluted threefold in the injection solvent 7:3 methanol:acetonitrile with 10 mM ammonium formate immediately before analysis. The injection solvent alone without any lipids was used as the ‘blank’ sample.

Mass spectrometry data were acquired for 3 min by flow injection (that is, no chromatographic separation). Briefly, 8 μl of diluted lipid extract stock solution delivered to the jet stream technology ion source (AJS) source of an Agilent 6495C Triple Quadrupole mass spectrometer. Multiple reaction monitoring methods were organized into 11 methods on the basis of the ten main lipid classes based on the LipidMaps database; see Extended Data Fig. 9d for total *n* of lipids screened and Supplementary Data 17 for individual species. TAGs were divided into two separate methods on the basis of fatty acid neutral loss residues.

Statistical analysis was performed using the EdgeR package^69^. EdgeR uses a generalized linear model to identify differentially expressed lipids. The generalized linear model is based on the negative binomial distribution that incorporates the blank with a dispersion term using the common dispersion method^70^. This allows it to model the technical and biological variability. This method was previously described in detail in ref. ^68^. Significant lipids were chosen on the basis of a false discovery rate value <0.1 (ref. ^71^).

### Whole-mount brain immunofluorescence

A standard protocol was used for fixation and staining. In brief, adult fly brains were dissected in cold PBS and fixed in 4% paraformaldehyde (v/v) for 50 min at room temperature. Brains were washed and permeabilized in PBS-0.1% Triton-X (PBST; 3×, 10 min). Samples were blocked in PBST-5% NGS at room temperature for 1 h, then incubated for 24–48 h at 4 °C with 1° antibody (1:25 mouse anti-repo, DSHB 8D12; 1:20 rat anti-elav, DSHB 7E8A10). Brains were washed in PBST then incubated with fluorescently conjugated 2° antibody for 1 h at room temperature. For AP1 activity (all genotypes containing *TRE-*dsRed) and tdTomato, endogenous fluorophore luminescence was measured without additional antibody staining. Brains were counterstained with Hoechst (0.10 mg ml^−1^ in PBS) for 15 min, cleared in mounting media (20 mM Tris pH 8.0, 0.5% *N*-propyl gallate, 80% glycerol, PBS), mounted in mounting media and cover slipped. Brains were imaged by confocal microscopy (Leica DM 6000 CS) with identical laser power and gain settings across experiments. Images were acquired throughout the full brain at 2 μM steps at 1,024 × 1,024 resolution by ×20 (dry) or ×63 (oil) objectives.

For BODIPY, brains were dissected and fixed as above then incubated for 24–48 h at room temperature in 1:250 dilution of 10 mg ml^−1^ BODIPY 493/503 (Invitrogen D3922) prepared in NGS. Brains were washed in PBST, counterstained with Hoechst and prepared for imaging as above.

For DHE, fly brains were rapidly dissected in cold Schneider’s medium, incubated in 60 μM DHE (ThermoFisher, catalogue no. D11347) for 7 min at room temperature shaking. Brains were washed in Schneider’s medium (2×, 5 min) then PBS (1×, 5 min), mounted in mounting media and imaged immediately (excitation 488 nm, emission 515–656 nm).

Fiji v.2.0 was used for analysing all images. For *TRE*-dsRed quantification, dsRed was measured in Fiji as raw integrated density in scaled images of the *z* stacked brain. For BODIPY 493/503 quantification, background was first subtracted from scaled images of the *z* stacked brains. Automatic thresholding was applied and Analyze Particles (Analyze>Analyze Particles) was used to determine the number, average size and total area of BODIPY^+^ droplets.

### Whole-mount brain immunohistochemistry for SA-β-Gal activity

A protocol was adapted^72^ for staining in fixed dissected whole *Drosophila* brains. In brief, adult fly brains were dissected in cold PBS and fixed in 2% paraformaldehyde and 0.2% glutaraldehyde (v/v) for 30 min at room temperature. Brains were washed in PBS (3×, 5 min) then incubated in 150 μl of X-Gal staining solution (40 mM citric acid phosphate buffer, 5 mM potassium hexanocyanoferrate(II) trihydrate, 5 mM potassium hexanocyanoferrate(III), 150 mM NaCl, 2 mM MgCl_2_-6H_2_O, 2.44 mM x-Gal) at 37 °C in the dark shaking (300 rpm) for a predetermined time on the basis of genotype (roughly 12–24 h). Brains were washed in PBS (3×, 5 min) and cleared in mounting media as above overnight. Brains were imaged on APO16 microscope and staining was quantified in Fiji v.2.0 by converting to a red, green and blue stack and measuring area and median value in the red channel only. Inverted density was calculated by subtracting median grey value from 255 and normalized to controls processed in parallel to account for variability across experiments.

### Western immunoblot

Fly brains were rapidly dissected in cold PBS (*n* = 8 brains per biological replicate), then homogenized in 5 μl of sample buffer per brain (1× Laemmli Buffer (Bio-Rad, catalogue no. 161-0737), 1× cOmplete mini EDTA-free protease inhibitor cocktail, 1 mM phenylmethylsulfonyl fluoride (Sigma, catalogue no. P7626), 50 μl β-mercaptoethanol (BME): Sigma, catalogue no. M6250). Samples were denatured (98 °C for 3 min) before loading onto 4–12% Bis-Tris gel. Volume equivalent of one brain per sample was run in 1× MES buffer, transferred to 0.45 μM nitrocellulose membrane overnight by electrophoresis. Membranes were stained by Ponceau S to confirm transfer. Membranes were blocked in 3% bovine serum albumin in 1× Tris-buffered saline, 0.1% Tween 20 detergent, incubated in primary antibody overnight at 4 °C (1:200 mouse anti-γH2Av, DSHB UNC93-5.2.1; 1:2,000 mouse anti-tubulin, DSHB AA4.3). Blots were incubated with 1:5,000 dilution of species-appropriate HRP-conjugated secondary antibody for 1 h at room temperature, then detected by ECL (Cytiva (formerly GE Healthcare Life Sciences), catalogue no. RPN2232) using an Amersham Imager 600. Quantification was performed in Fiji by region of interest. Sample protein was normalized to the loading control alpha tubulin. Mammalian cells were lysed in modified RIPA buffer and blotted using standard techniques as previously described^73^ using an antibody to JUN (1:1,000; Cell Signaling Technology, catalogue no. 9165).

### Cell proliferation by EdU labelling

Flies were maintained on 0.2 mM EdU food from eclosion through dissection. EdU staining was performed according to the manufacturer’s protocol (Click-iT EdU Imaging Kit; ThermoFisher, catalogue no. mp10338). In brief, brains were dissected and fixed as above for immunohistochemistry. Following permeabilization, brains were incubated in Click-iT reaction mixture overnight at 4 °C. Brains were washed, counterstained with Hoechst, cleared, mounted and imaged as above.

### Mitochondrial function assay

The ratio of ATP/cytotoxicity was determined using the Promega Mitochondrial ToxGlo Assay (G8001). The manufacturer’s instructions were followed. Assay lysates consisted of individual dissected fly brains (*n* = 3–4 brains per genotype or condition) across a minimum of three experiments. Samples were always normalized to parallel processed controls.

### mtDNA PCR

A protocol was adapted for measuring mtDNA in adult fly heads^34^. For head collection, whole flies were anaesthetized by CO_2_, frozen by submersion in liquid nitrogen, then vortexed to separate heads from bodies. Total cellular DNA was extracted by homogenizing five heads (per replicate) in 30 µl working solution (10 mM Tris-HCl, pH 8.0, 1 mM EDTA, 0.1% Triton-X-100 and 10 μg ml^−1^ protease K). Samples were then incubated at 37 °C for 60 min, and followed by inactivation of protease K at 95 °C for 10 min. Head cuticles were pelleted by centrifuging samples at 12,000*g* for 10 min at room temperature. Supernatants were transferred into a new tube, before measuring DNA concentration by Nanodrop. mtDNA was quantified using nuclear DNA (GAPDH) as control in real-time quantitative PCR (qPCR). Primer sequences: mtDNA-F1, GAATTAGGACATCCTGGAGC and mtDNA-R1, GCACTAATCAATTTCCAAATCC; GAPDH-F1, GACGAAATCAAGGCTAAGGTCG and GAPDH-R1, AATGGGTGTCGCTGAAGAAGTC.

### Real-time qPCR

Total RNA was isolated from fly brains or heads (*n* = 8–20 per replicate) by RNeasy Mini Kit (Qiagen, catalogue no. 74104), with on-column removal of genomic DNA (Qiagen, catalogue no. 79254). Complementary DNA (cDNA) was prepared from total RNA (Applied Biosystems, catalogue no. 4368814) then quantified by Qubit ssDNA Assay (Invitrogen, catalogue no. Q10212). Real-time qPCR reactions were set up using Fast SYBR Green reagents (ThermoFisher, catalogue no. 4385612) in 384-well plates with 20 ng of cDNA per reaction and analysed on a ViiA 7 Real-Time PCR System (Applied Biosystems). Relative expression was determined using the ∆∆CT method. For each sample, mean CT values were determined from 2–3 technical replicates. ∆CT was determined relative to the housekeeping gene, *β-tubulin*. ∆∆CT was then calculated as fold change relative to the control group. Real-time qPCR primers were sourced from FlyPrimerBank^74^ or previous publications, BLASTd against the *Drosophila* genome for specificity and optimized by serial dilution curve and melt curve analysis. See Supplementary Table 2 for primer sequences.

### Mammalian cell culture

IMR90 primary human fibroblasts (ATCC CCL-186) were grown at 37 °C, 3.5% O_2_, 5% CO_2_, in Dulbecco’s modified Eagle’s medium (Gibco, catalogue no. 10313-121) with 10% FBS (Corning, catalogue no. 35-011-CV), 1% penicillin–streptomycin (Gibco, catalogue no. 15140-122) and 2 mM glutamine (Gibco, catalogue no. 25030-081). Cultures were checked routinely for mycoplasma contamination. Irradiation senescence was induced by 20 Gray of X-ray irradiation of 20–30% confluent cells. Cells were split after returning to confluence 3 days after irradiation. On days 4 and 7 after irradiation, cells were transfected with a pool of four small-interfering RNA against JUN (Dharmacon siGENOME) or non-targeting control (siNTC #3, Dharmacon siGENOME) to a final concentration of 100 nM with 0.8% Dharmafect reagent following the manufacturer’s protocol. Medium was changed 18–20 h after each transfection. Medium from days 8–10 after irradiation, or from normal proliferating IMR90 cells cultured in parallel, was collected, centrifuged at 500*g* for 3 min to remove whole cells and large debris, then added to 20–30% confluent proliferating IMR90 cells plated on 96-well imaging plates (Perkin Elmer, catalogue no. 6055302) for 48 h. Cells were fixed in 10% neutral buffered formalin (Epredia, catalogue no. 9400-1) and stained with 500 µg ml^−1^ DAPI and 5 µg ml^−1^ BODIPY 493/503 (Cayman, catalogue no. 25892) in PBS. Automated imaging of cells was done on a Nikon Ti2 microscope and images were analysed in NIS Elements.

### Statistical analysis

Statistical analysis and data visualization were performed in the R Environment using RStudio with base R and packages as indicated including with tidyverse (dplyr, ggplot2), ggrepel, cowplot, ggsurvplot. No statistical method was used to predetermine sample sizes; standard sample sizes for *Drosophila*, pooled across two or three independent experiments, were used. See the Source Data files for data and statistical reporting corresponding to each main and Extended Data figure.

### Reporting summary

Further information on research design is available in the Nature Portfolio Reporting Summary linked to this article.

## Online content

Any methods, additional references, Nature Portfolio reporting summaries, source data, extended data, supplementary information, acknowledgements, peer review information; details of author contributions and competing interests; and statements of data and code availability are available at 10.1038/s41586-024-07516-8.

### Supplementary information

Supplementary Information This file contains a guide to the Supplementary files; legends for the Supplementary Video, Data files and Tables 1 and 2.

Reporting Summary

Supplementary Fig. 1

Supplementary Data files 1–17

Supplementary Video 1 LDs accumulate in glia with age. Confocal microscopy of a 40-day-old fly brain; BODIPY^+^ LDs (green) are present throughout the central brain and localize to glial processes (red). Genotype is *repo*-GS>*UAS*-tdTomato^CYTO^.

Supplementary Video 2 LDs are absent from AP1^+^ glia. Confocal microscopy of a 40-day-old fly brain; BODIPY^+^ LDs (green) do not colocalize with AP1^+^ glial (red). Genotype is *TRE*-dsRed.

### Source data

Source Data Fig. 1

Source Data Fig. 2

Source Data Fig. 3

Source Data Fig. 4

Source Data Fig. 5

Source Data Extended Data Fig. 1

Source Data Extended Data Fig. 2

Source Data Extended Data Fig. 3

Source Data Extended Data Fig. 4

Source Data Extended Data Fig. 5

Source Data Extended Data Fig. 6

Source Data Extended Data Fig. 7

Source Data Extended Data Fig. 8

Source Data Extended Data Fig. 9

Source Data Extended Data Fig. 10

## Data Availability

RNA-seq data that support the findings of this study have been deposited in the Gene Expression Omnibus (accession codes GSE263926, GSE263927, GSE263928, GSE263929). Raw and processed lipidomic data are available on GitHub: https://github.com/chopralab/drosophila_brain_lipidomics_Byrns_et_all. Source data are provided with this paper.
