# Author Correction: Senescent glia link mitochondrial dysfunction and lipid accumulation

**DOI:** 10.1038/s41586-024-07904-0

**Published:** 2024-08-07

**Authors:** China N. Byrns, Alexandra E. Perlegos, Karl N. Miller, Zhecheng Jin, Faith R. Carranza, Palak Manchandra, Connor H. Beveridge, Caitlin E. Randolph, V. Sai Chaluvadi, Shirley L. Zhang, Ananth R. Srinivasan, F. C. Bennett, Amita Sehgal, Peter D. Adams, Gaurav Chopra, Nancy M. Bonini

**Affiliations:** 1 Medical Scientist Training Program, Perelman School of Medicine, University of Pennsylvania, Philadelphia, PA USA; 2 Department of Biology, University of Pennsylvania, Philadelphia, PA USA; 3 Neuroscience Graduate Group, Perelman School of Medicine, University of Pennsylvania, Philadelphia, PA USA; 4 Cancer Genome and Epigenetics Program, Sanford Burnham Prebys Medical Discovery Institute, La Jolla, CA USA; 5 Department of Chemistry, Purdue University, West Lafayette, IN USA; 6 Howard Hughes Medical Institute and Chronobiology and Sleep Institute, Perelman School of Medicine at the University of Pennsylvania, Philadelphia, PA USA; 7 Department of Psychiatry, Perelman School of Medicine, University of Pennsylvania, Philadelphia, PA USA; 8 Division of Neurology, Children’s Hospital of Philadelphia, Philadelphia, PA USA; 9 Purdue Institute for Integrative Neuroscience, Purdue University, West Lafayette, IN USA; 10 Purdue Institute for Drug Discovery, Purdue University, West Lafayette, IN USA; 11 Purdue Center for Cancer Research, Purdue University, West Lafayette, IN USA; 12 Purdue Institute of Inflammation, Immunology and Infectious Disease, Purdue University, West Lafayette, IN USA

**Keywords:** Cellular neuroscience, Glial biology, Neural ageing, Senescence

Correction to: *Nature* 10.1038/s41586-024-07516-8 Published online 5 June 2024

In the version of the article initially published, in Fig. 1h, the lower label originally read “AP1^+^ glia (dsRed^neg^GFP^+^)” and has now been corrected to “AP1^+^ glia (dsRed^pos^GFP^+^)” in the HTML and PDF versions of the article.

