# Supplementary material for: Senescent glia link mitochondrial dysfunction and lipid accumulation
Source: Nature. 2024 Jun 5;630(8016):475–83. doi: 10.1038/s41586-024-07516-8 (PMC11168935; doi:10.1038/s41586-024-07516-8)
Supplement: Supplementary file 1 — This file contains a guide to the Supplementary files; legends for the Supplementary Video, Data files and Tables 1 and 2. [file 41586_2024_7516_MOESM1_ESM.pdf]

nature portfolio

https://doi.org/10.1038/s41586-024-07516-8

Supplementary information

Senescent glia link mitochondrial dysfunction and lipid accumulation

In the format provided by the authors and unedited

SUPPLEMENTARY INFORMATION GUIDE TO:

Senescent glia link mitochondrial dysfunction and lipid accumulation

China N. Byrns[1,2], Alexandra E. Perlegos[3], Karl Miller[4], Zhecheng Jin[2], Faith R. Carranza[2], Palak Manchandra[5], Connor H. Beveridge[5], Caitlin E. Randolph[5], V Sai Chaluvadi[1,3], Shirley L. Zhang[6], Ananth R. Srinivasan[2], F.C. Bennett[7,8], Amita Sehgal[6], Peter D. Adams[4], Gaurav Chopra[5,9,10,11,12], Nancy M. Bonini[2,3*]

Affiliations:
[1]Medical Scientist Training Program, Perelman School of Medicine, University of Pennsylvania, Philadelphia, PA, USA
[2]Department of Biology, University of Pennsylvania, Philadelphia, PA, USA
[3]Neuroscience Graduate Group, Perelman School of Medicine, University of Pennsylvania, Philadelphia, PA, USA
[4]Cancer Genome and Epigenetics Program, Sanford Burnham Prebys Medical Discovery Institute, La Jolla, CA, USA
[5]Department of Chemistry, Purdue University, West Lafayette, IN, USA
[6]Howard Hughes Medical Institute and Chronobiology and Sleep Institute, Perelman School of Medicine at the University of Pennsylvania, Philadelphia, PA, USA
[7]Department of Psychiatry, Perelman School of Medicine, University of Pennsylvania, Philadelphia, PA, USA
[8]Division of Neurology, Children's Hospital of Philadelphia, Philadelphia, PA, USA
[9]Purdue Institute for Integrative Neuroscience, Purdue University, West Lafayette, IN, USA
[10]Purdue Institute for Drug Discovery, Purdue University, West Lafayette, IN, USA
[11]Purdue Center for Cancer Research, Purdue University, West Lafayette, IN, USA
[12]Purdue Institute of Inflammation, Immunology and Infectious Disease, Purdue University, West Lafayette, IN, USA

Supplementary Video 1: Lipid droplets accumulate in glia with age.
Confocal microscopy of a 40 d old fly brain; BODIPY+ lipid droplets (green) are present throughout the central brain and localize to glial processes (red). Genotype is *repo*-GS>*UAS*-tdTomato^{CYTO}.

Supplementary Video 2: Lipid droplets are absent from AP1+ glia.
Confocal microscopy of a 40 d old fly brain; BODIPY+ lipid droplets (green) do not colocalize with AP1+ glial (red). Genotype is *TRE*-dsRed.

Supplementary Data 1: Table of significantly differentially expressed genes between FACS-isolated and bulk RNA-sequenced neurons (dsRed^{neg}GFP^{neg}), AP1+ glia (dsRed+GFP+), and AP1neg glia (dsRed^{neg}GFP+) from 40 d old brains. Genotype is *repo*-GAL4>*TRE*-dsRed,*UAS*-GFP.

Supplementary Data 2: Table of Reactome pathways enriched among differentially expressed genes between FACS-isolated AP1+ glia compared to neurons or AP1neg glia (40 d). Genotype is *repo*-GAL4>*TRE*-dsRed,*UAS*-GFP.

Supplementary Data 3: Table of significantly differentially expressed genes between FACS-isolated and bulk RNA-sequenced neurons (dsRed^{neg}GFP^{neg}) from aged (40 d) vs young (5 d) brains. Genotype is *repo*-GAL4>*TRE*-dsRed,*UAS*-GFP.

Supplementary Data 4: Table of GO and Reactome pathways enriched among differentially expressed genes between FACS-isolated and bulk RNA-sequenced neurons from aged (40 d) vs young (5 d) brains. Genotype is *repo*-GAL4>*TRE*-dsRed,*UAS*-GFP.

Supplementary Data 5: Table of significantly differentially expressed genes in whole brains of 10 d flies with neuronal knockdown of ND42 or ND30, compared to UAS-mCherry-RNAi control. Genotypes are *TRE*-dsRed;*elav*-GS>*UAS*-ND42-RNAi, *TRE*-dsRed;*elav*-GS>*UAS*-ND30-RNAi or *TRE*-dsRed;*elav*-GS>*UAS*-mCherry-RNAi.

Supplementary Data 6: Table of GO and Reactome pathways enriched among differentially expressed genes in whole brains of 10 d flies with neuronal knockdown of ND42 or ND30, compared to UAS-mCherry-RNAi control. Genotypes are *TRE*-dsRed;*elav*-GS>*UAS*-ND42-RNAi, *TRE*-dsRed;*elav*-GS>*UAS*-ND30-RNAi or *TRE*-dsRed;*elav*-GS>*UAS*-mCherry-RNAi.

Supplementary Data 7: Table of significantly differentially expressed genes shared by with neuronal knockdown of ND42 or ND30 relative to UAS-mCherry-RNAi control (whole brains; 10 d age). Genotypes are *TRE*-dsRed;*elav*-GS>*UAS*-ND42-RNAi, *TRE*-dsRed;*elav*-GS>*UAS*-ND30-RNAi or *TRE*-dsRed;*elav*-GS>*UAS*-mCherry-RNAi.

Supplementary Data 8: Table of significantly differentially expressed genes in FACS-isolated and bulk RNA-sequenced dsRed+ and dsRedneg cells from 10 d brains with neuronal knockdown of NP15.6 or ND42. Genotypes are *TRE*-dsRed;*elav*-GS>*UAS*-ND42-RNAi or *TRE*-dsRed;*elav*-GS>*UAS*-NP15.6-RNAi.

Supplementary Data 9: Table of GO and Reactome pathways enriched in FACS-isolated and bulk RNA-sequenced dsRed+ and dsRedneg cells from 10 d brains with neuronal knockdown of NP15.6 or ND42. Genotypes are *TRE*-dsRed;*elav*-GS>*UAS*-ND42-RNAi or *TRE*-dsRed;*elav*-GS >*UAS*-NP15.6-RNAi.

Supplementary Data 10: Table of significantly differentially expressed genes in whole brains from 42 d old flies with intermittent glial AP1 blockade (RU486 1d/wk) by *UAS*-dFosDN or *UAS*-puckered relative to UAS-GFP controls. Genotypes are *repo*-GS>*UAS*-dFos^{DN}, *repo*-GS>*UAS*-puckered, or *repo*-GS>*UAS*-GFP.

Supplementary Data 11: Table of GO and Reactome pathways enriched in whole brains from 42 d old flies with intermittent glial AP1 blockade (RU486 1d/wk) by *UAS*-dFosDN or *UAS*-puckered, relative to *UAS*-GFP controls. Genotypes are *repo*-GS>*UAS*-dFos^{DN}, *repo*-GS>*UAS*-puckered, or *repo*-GS>*UAS*-GFP.

Supplementary Data 12: Normalized counts data for FACS-isolated and bulk RNA-sequenced neurons (dsRed^{neg}GFP^{neg}), AP1+ (dsRed+GFP+) and AP1^{neg} glia (dsRed^{neg}GFP+) from 40 d old brains. Genotype is *repo*-GAL4>*TRE*-dsRed,*UAS*-GFP.

Supplementary Data 13: Normalized counts data for FACS-isolated and bulk RNA-sequenced 5d and 40d old neurons (dsRed^{neg}GFP^{neg}). Genotype is *repo*-GAL4>*TRE*-dsRed,*UAS*-GFP.

Supplementary Data 14: Normalized counts data for bulk RNA-sequenced brains from 10 d old flies with neuronal knockdown of ND42 or ND30, compared to *UAS*-mCherry-RNAi control. Genotypes are *TRE*-dsRed;*elav*-GS>*UAS*-ND42-RNAi, *TRE*-dsRed;*elav*-GS>*UAS*-ND30-RNAi or *TRE*-dsRed;*elav*-GS>*UAS*-mCherry-RNAi.

Supplementary Data 15: Normalized counts data for FACS-isolated and bulk RNA-sequenced dsRed+ and dsRedneg cells from 10 d brains with neuronal knockdown of NP15.6 or ND42. Genotypes are *TRE*-dsRed;*elav*-GS>*UAS*-ND42-RNAi or *TRE*-dsRed;*elav*-GS>*UAS*-NP15.6-RNAi.

Supplementary Data 16: Normalized counts data for bulk RNA-sequenced brains from 7 d and 42 d old flies with intermittent glial AP1 blockade (RU486 1d/wk) by UAS-dFosDN or UAS-puckered or UAS-GFP controls. Genotypes are *repo*-GS>*UAS*-dFos^{DN}, *repo*-GS>*UAS*-puckered, or *repo*-GS>*UAS*-GFP.

Supplementary Data 17: List of lipids screened for lipodomic analyses of whole brains and FACS-isolated cells, based on 1324 multiple reaction monitoring (MRM) transitions to identify lipid species. Lipid types include acyl carnities (AC), cholesterol esters (CE), ceramides (CER), free fatty acids (FFA), phosphatidylcholine (PC) and sphingomyelin (SM), phosphatidylethanolamine (PE), phosphatidylglycerol (PG), phosphatidylinositol (PI), triacylglycerides (TAG).

Supplementary Table 1. *Drosophila* genotype information

Name in this paper	ID**	Full Genotype	Reference	Notes
w^{1118}	RRID:BDSC_5905	*w[1118]*	-	-
UAS-GFP	-	*yw; UAS-mCD8-GFP/CyO;*	-	-
UAS-dFos^{DN}	RRID:BDSC_ 7214	*w[1118]; P{w[+mC]=UAS-Fra.Fbz}5*		
UAS-dFos^{DN}	RRID_BDSC_7215	*y[1] w[1118]; P{w[+mC]=UAS-Fra.Fbz}7*	-	-
TRE-dsRed	RRID:BDSC_59012	*w[*]; P{y[+t7.7] w[+mC]=TRE-DsRedT4}attP16*	Chatterjee & Bohmann, 2012.	Courtesy of Marc Freeman
repo-GAL4	RRID_BDSC_7415	*w[1118]; P{w[+m*]=GAL4}repo/TM3, Sb[1]*	-	
repo-GS (geneSwitch)	-	*w^{1118};;P{repoGS}attP154*	-	Courtesy of Herve Tricoire; referred to as *repoGS2301*
elav-GS (geneSwitch)	-	*y*w*;;elav-geneSwitch*	-	-
TRE-dsRed,*UAS* -GFP	-	*w[*]/w1118; TREdsRed,UAS-mcd8-GFP/SM6A,Cy*	This paper	Recombinant made for this study
TRE-dsRed;*elav*-GS	-	*w[*]; P{y[+t7.7] w[+mC]=TRE-DsRedT4}attP16;elav-GeneSwitch*	This paper	Recombinant made for this study
repo-GS,*UAS*-dFos^{DN}	-	*w[1118]/w[*]; P{repoGeneSwitch}attP40, P{w[+mC]=UAS-Fra.Fbz}5*	This paper	Recombinant made for this study
UAS-puckered	-	*y-w-;;UAS-puckered*	-	Courtesy of Marc Freeman
UAS-mCherry-RNAi	RRID_BDCS_ 35785	*y[1] sc[*] v[1]; P{y[+t7.7] v[+t1.8]=VALIUM20-mCherry}attP2*		Control RNAi line
UAS-ND15-RNAi	RRID:BDSC_55180	*y[1] sc[*] v[1] sev[21]; P{y[+t7.7] v[+t1.8]=TRiP.HMC03861}attP40*		Experimental RNAi line (inner mito gene)
UAS-ATPsynC-RNAi	RRID:BDSC_57705	*y[1] sc[*] v[1] sev[21]; P{y[+t7.7] v[+t1.8]=TRiP.HMC04894}attP40*		Experimental RNAi line (inner mito gene)
UAS-ND42-RNAi	RRID:BDSC_32998	*y[1] sc[*] v[1] sev[21]; P{y[+t7.7] v[+t1.8]=TRiP.HMS00798}attP2*		Experimental RNAi line (inner mito gene)
UAS-ND75-RNAi	RRID:BDSC_33910	*y[1] sc[*] v[1] sev[21]; P{y[+t7.7] v[+t1.8]=TRiP.HMS00853}attP2 (May be segregating TM3, Sb[1].)*		Experimental RNAi line

				(inner mito gene)
UAS-NP15.6-RNAi	RRID:BDSC_36672	*y[1] sc[*] v[1] sev[21]; P{y[+t7.7] v[+t1.8]=TRiP.HMS01560}attP2*		Experimental RNAi line (inner mito gene)
UAS-ND-30-RNAi	RRID:BDSC_44535	*y[1] v[1]; P{y[+t7.7] v[+t1.8]=TRiP.HMC02929}attP40*		Experimental RNAi line (inner mito gene)
UAS-COX5A-RNAi	RRID:BDSC_58282	*y[1] v[1]; P{y[+t7.7] v[+t1.8]=TRiP.HMJ22367}attP40*		Experimental RNAi line (inner mito gene)
UAS-marf-RNAi	RRID:BDSC_55189	*y[1] sc[*] v[1] sev[21]; P{y[+t7.7] v[+t1.8]=TRiP.HMC03883}attP40*		Experimental RNAi line (mito gene)
UAS-parkin-RNAi	RRID:BDSC_37509	*y[1] sc[*] v[1] sev[21]; P{y[+t7.7] v[+t1.8]=TRiP.HMS01651}attP40*		Experimental RNAi line (mito gene)
UAS-pink1-RNAi	RRID:BDSC_41671	*y[1] sc[*] v[1] sev[21]; P{y[+t7.7] v[+t1.8]=TRiP.HMS02204}attP2*		Experimental RNAi line (mito gene)
UAS-Opa1-RNAi	RRID:BDSC_32358	*y[1] sc[*] v[1] sev[21]; P{y[+t7.7] v[+t1.8]=TRiP.HMS00349}attP2*		Experimental RNAi line (mito gene)
UAS-Lip4	RRID_BDSC_ 67142	*y[1] w[*]; PBac{y[+mDint2] w[+mC]=UAS-Lip4.L}VK00037*	-	-
UAS-bmm	RRID_BDSC_ 76600	*w[*]; P{w[+mC]=UAS-bmm.cGa}2*	-	-
UAS-SREBP^{DN}	RRID_BDSC_ 8245	*y[1] w[1118]; P{w[+mC]=UAS-SREBP.P450}3*	-	-
UAS-tdTom^{CYTO}	RRID_BDSC_ 92758	*w[1118]; PBac{y[+mDint2] w[+mC]=UAS-tdTom.K}VK00016*	-	-

Supplementary Table 2. Real-time qPCR primer information

Target gene	Forward primer (5' to 3')	Reverse primer (5' to 3')	Concentration	Reference
ND42	AGTACAGCGTGTTCAATGCTATT	CCACACAGATCACCTTGGAGT	5μM	FlyPrimerBank: PP19590
NP15.6	CAGCTTCTACTGGGCCTACCT	GTAGTTGGGGCTGACCAAATC	5μM	FlyPrimerBank: PP34270
ND15	ACTGATGCGCTTCCATGCAAT	CTTGTCCCTTACGCTCCCC	5μM	FlyPrimerBank: PP10646
ND75	AACTCGGATTTGACCCGCAAG	CCGTGTAGTTAATGTCCGTGAA	5μM	FlyPrimerBank: PP34007
ND30	AAGGCGGATAAGCCCACTGTC	CCCTCTGGCGCAATAAGCA	5μM	This paper
ATPSynC	CCACAGATCAGGTCATTCCAGA	CGAATACTGTTCCGATACCAGC	5μM	FlyPrimerBank: PP22521
COX5A	GCATCCCAACCATCGAGGAA	AGGCATCGTACACGGACTTC	5μM	FlyPrimerBank: PD44440
parkin	GAAGCCTCCAAGCCTCTAAATG	ACGGACTCTTTCTTCATCGGT	5μM	FlyPrimerBank: PP33010
pink1	AAGCGAGGCTTTCCCCTAC	GCACTACATTGACCACCGATTT	5μM	FlyPrimerBank: PP23832
opa1	CGAGAAAAGCTGTTGTCTACTCC	GATGGCTGGTGATGCCACA	5μM	FlyPrimerBank: PP2522
marf	AGTGCCAGGAATCGGAATTGG	CCTTTAGCTCCTTGGTGAGGAA	5μM	This paper
Irbp	AGTTCATCACGTTGTCAAGAGC	TACGATCGGACAGGATTTCG	5μM	FlyPrimerBank: PB60172
Dop1R	GCATCTTCCTATCGGTGCTGA	AGATCCGCAATCGCTAACGAG	5μM	FlyPrimerBank: PP20391
Vglut	AAGTTCCCGGCCAACAAGATA	CTCACGCAAATCACCACATGA	5μM	FlyPrimerBank: PP30371
β-tubulin	CATCCAAGCTGGTCAGTG	GCCATGCTCATCGGAGAT	5μM	McGurk et al., 2018
