# Supplementary material for: Senescent glia link mitochondrial dysfunction and lipid accumulation
Source: Nature. 2024 Jun 5;630(8016):475–83. doi: 10.1038/s41586-024-07516-8 (PMC11168935; doi:10.1038/s41586-024-07516-8)
Supplement: Supplementary file 2 — Reporting Summary [file 41586_2024_7516_MOESM2_ESM.pdf]

nature portfolio

Corresponding author(s): Nancy M. Bonini

Last updated by author(s): Apr 24, 2024

Reporting Summary

Nature Portfolio wishes to improve the reproducibility of the work that we publish. This form provides structure for consistency and transparency in reporting. For further information on Nature Portfolio policies, see our Editorial Policies and the Editorial Policy Checklist.

Statistics

For all statistical analyses, confirm that the following items are present in the figure legend, table legend, main text, or Methods section.

n/a	Confirmed	
☐	☒	The exact sample size (n) for each experimental group/condition, given as a discrete number and unit of measurement
☐	☒	A statement on whether measurements were taken from distinct samples or whether the same sample was measured repeatedly
☐	☒	The statistical test(s) used AND whether they are one- or two-sided<br>*Only common tests should be described solely by name; describe more complex techniques in the Methods section.*
☒	☐	A description of all covariates tested
☐	☒	A description of any assumptions or corrections, such as tests of normality and adjustment for multiple comparisons
☐	☒	A full description of the statistical parameters including central tendency (e.g. means) or other basic estimates (e.g. regression coefficient) AND variation (e.g. standard deviation) or associated estimates of uncertainty (e.g. confidence intervals)
☐	☒	For null hypothesis testing, the test statistic (e.g. F, t, r) with confidence intervals, effect sizes, degrees of freedom and P value noted<br>*Give P values as exact values whenever suitable.*
☒	☐	For Bayesian analysis, information on the choice of priors and Markov chain Monte Carlo settings
☐	☒	For hierarchical and complex designs, identification of the appropriate level for tests and full reporting of outcomes
☐	☒	Estimates of effect sizes (e.g. Cohen's d, Pearson's r), indicating how they were calculated

Our web collection on statistics for biologists contains articles on many of the points above.

Software and code

Policy information about availability of computer code

Data collection	No custom software or analyses were used.
Data analysis	No custom software or analyses were used.

For manuscripts utilizing custom algorithms or software that are central to the research but not yet described in published literature, software must be made available to editors and reviewers. We strongly encourage code deposition in a community repository (e.g. GitHub). See the Nature Portfolio guidelines for submitting code & software for further information.

Data

Policy information about availability of data

All manuscripts must include a data availability statement. This statement should provide the following information, where applicable:

- Accession codes, unique identifiers, or web links for publicly available datasets
- A description of any restrictions on data availability
- For clinical datasets or third party data, please ensure that the statement adheres to our policy

Source data are provided with this paper. RNA-sequencing data that support the findings of this study have been deposited in the Gene Expression Omnibus (Accession codes: GSE263926, GSE263927, GSE263928, GSE263929). Raw and processed lipidomic data are available on GitHub: https://github.com/chopralab/drosophila_brain_lipidomics_Byrns_et_all

Research involving human participants, their data, or biological material

Policy information about studies with human participants or human data. See also policy information about sex, gender (identity/presentation), and sexual orientation and race, ethnicity and racism.

Reporting on sex and gender	n/a
Reporting on race, ethnicity, or other socially relevant groupings	n/a
Population characteristics	n/a
Recruitment	n/a
Ethics oversight	n/a

Note that full information on the approval of the study protocol must also be provided in the manuscript.

Field-specific reporting

Please select the one below that is the best fit for your research. If you are not sure, read the appropriate sections before making your selection.

☒ Life sciences ☐ Behavioural & social sciences ☐ Ecological, evolutionary & environmental sciences

For a reference copy of the document with all sections, see nature.com/documents/nr-reporting-summary-flat.pdf

Life sciences study design

All studies must disclose on these points even when the disclosure is negative.

Sample size	Sample size was determined based on precedent by our lab and others, specific experimental parameters and readout, and considering downstream statistical tests. Sample size cutoff of n<15 was generally used for non-parametric tests and n>15 for parametric tests, along with testing for homogeneity of variance (Levene's test) and normality (Shapiro-Wilks test).
Data exclusions	No data were excluded from analyses.
Replication	All data shown are the result of a minimum of two independent experiments, based on positive findings from an initial pilot study.
Randomization	Male sibling flies were randomly assigned to experimental conditions. For experiments with non-sibling flies (i.e: different genetic background), flies were age-matched and handled in parallel.
Blinding	Samples were given non-identifying IDs so that experimentation, data acquisition, and quantification was performed blind to sample identity.

Reporting for specific materials, systems and methods

We require information from authors about some types of materials, experimental systems and methods used in many studies. Here, indicate whether each material, system or method listed is relevant to your study. If you are not sure if a list item applies to your research, read the appropriate section before selecting a response.

Materials & experimental systems

n/a	Involved in the study	
☐	☒	Antibodies
☐	☒	Eukaryotic cell lines
☒	☐	Palaeontology and archaeology
☐	☒	Animals and other organisms
☒	☐	Clinical data
☒	☐	Dual use research of concern
☒	☐	Plants

Methods

n/a	Involved in the study	
☒	☐	ChIP-seq
☐	☒	Flow cytometry
☒	☐	MRI-based neuroimaging

Antibodies

Antibodies used	Primary antibodies:<br>Whole mount immunofluorescence:

mouse anti-repo (DSHB cat.8D12)
rat anti-elav (DSHB cat.7E8A10)

Western immunoblots:
mouse anti-gH2AV (DSHB cat. UNC93-5.2.1)
mouse anti-tubulin (DSHB cat. AA4.3
rabbit anti-JUN (Cell Signaling Technology cat. 9165)

For FACS-sorting of fixed cells:
mouse anti-gH2AV (DSHB cat. UNC93-5.2.1)

Secondary antibodies:
goat anti-mouse Alexafluor488 (ThermoFisher Scientific cat. A28175)
goat anti-rat Alexafluor647 (ThermoFisher Scientific cat. A-21247)
goat anti-mouse AlexaFluor647 (ThermoFisher Scientific cat. A-21235)

Validation	All antibodies used in this study are well-validated in prior work and were validated for use prior to study.<br>repo: well-established glial marker in Drosophila, see https://dshb.biology.uiowa.edu/8D12-anti-Repo<br>elav: well-established neuronal marker in Drosophila, see https://dshb.biology.uiowa.edu/8D12-anti-Repo<br>aTubulin: well-established, correct band size, see https://dshb.biology.uiowa.edu/AA4-3<br>gH2AV: well-established neuronal marker in Drosophila, see https://dshb.biology.uiowa.edu/UNC93-5-2-1<br>JUN: see https://www.cellsignal.com/products/primary-antibodies/c-jun-60a8-rabbit-mab/9165

Eukaryotic cell lines

Policy information about cell lines and Sex and Gender in Research

Cell line source(s)	IMR90 cells are human fibroblasts isolated from normal lung tissue derived from a 16-week old female; cells were obtained from ATCC (CCL-186).
Authentication	The cells were not authenticated.
Mycoplasma contamination	Cultures were checked routinely for mycoplasma contamination.
Commonly misidentified lines<br>(See ICLAC register)	IMR90 is not a commonly misidentified line.

Animals and other research organisms

Policy information about studies involving animals; ARRIVE guidelines recommended for reporting animal research, and Sex and Gender in Research

Laboratory animals	All animals were males.
Wild animals	This study did not involve wild animals.
Reporting on sex	All animals were males to control for sex-based differences in aging and lifespan.
Field-collected samples	This study did not involve field-collected samples.
Ethics oversight	n/a (all live animals were invertebrates, ethical approval and oversight not required).

Note that full information on the approval of the study protocol must also be provided in the manuscript.

Plants

Seed stocks	n/a
Novel plant genotypes	n/a
Authentication	n/a

Flow Cytometry

Plots

Confirm that:

☒ The axis labels state the marker and fluorochrome used (e.g. CD4-FITC).

☒ The axis scales are clearly visible. Include numbers along axes only for bottom left plot of group (a 'group' is an analysis of identical markers).

☒ All plots are contour plots with outliers or pseudocolor plots.

☒ A numerical value for number of cells or percentage (with statistics) is provided.

Methodology

Sample preparation	All work was performed in RNAse-free conditions. To create cell suspension for FACS-based sorting, adult fly brains (n=20 per biological replicate) were rapidly dissected in cold Schneider's media with 45 uM actinomycin D and stored on ice until dissections were complete. Brains were then washed in cold PBS (3x). A single cell suspension was achieved by enzymatic/ physical dissociation as follows: whole brains were incubated in dissociation buffer (300 ul activated papain, Worthington PAP2 LK003178 and 4.1 ul liberase, Roche 5401119001) at 25C at 1000 rpm for a total of 20 min. During incubation, at 5 and 10 min, tissue was gently homogenized by pipetting. At 15 min, the entire homogenate was passed through 25G 5/8 needle (7x). At 20 min, enzymatic activity was halted by the addition of cold Schneider's medium. Cells were then strained (35 uM filter), pelleted (800g, 7 min) and resuspended in cold Schneider's medium with actinomycin D and 2.5 ul RNAse inhibitor (Takara Recombinant RNase Inhibitor, 2313A). Cells were resuspended in 250 ul, counterstained with 5 uM DAPI (live/dead) and 50 nM syto60 (nuclear; ThermoFisher S11342) and sorted by the Penn Cytomics and Cell Sorting Facility using a BD FACS Aria II SORP (100 uM nozzle; purity).
Instrument	BD FACS Aria II SORP (100 uM nozzle; purity)
Software	FlowJo v.10.8.
Cell population abundance	For setting initial gating parameters, 20,000 cells were sorted of which 5486 cells passed all parameters (660/20 RedA, 450.50 Violet-A, FSC-HxFSC-W, SSC-HxSSC-W). From this population, 58 cells (0.3%) were AP1+ glia (dsRed+GFP), 1213 cells (6.1%) were AP1neg glia (dsRednegGFP+) and 3008 cells (15%) were neurons (dsRednegGFPneg). This abundance is representative of all samples.
Gating strategy	Dead cells were excluded through DAPI uptake. Doublets were excluded through FSC-H by FSC-W and SSC-H by SSC-W parameters. Nucleated cells were included by syto60. Glia were identified by GFP while neurons were GFP negative. AP1 activity was identified by dsRed. Gating strategy in Extended Data Fig.1c.

☒ Tick this box to confirm that a figure exemplifying the gating strategy is provided in the Supplementary Information.
