# Supplementary material for: Senescent glia link mitochondrial dysfunction and lipid accumulation
Source: Nature. 2024 Jun 5;630(8016):475–83. doi: 10.1038/s41586-024-07516-8 (PMC11168935; doi:10.1038/s41586-024-07516-8)
Supplement: Supplementary file 3 — Supplementary Fig. 1 [file 41586_2024_7516_MOESM3_ESM.pdf]

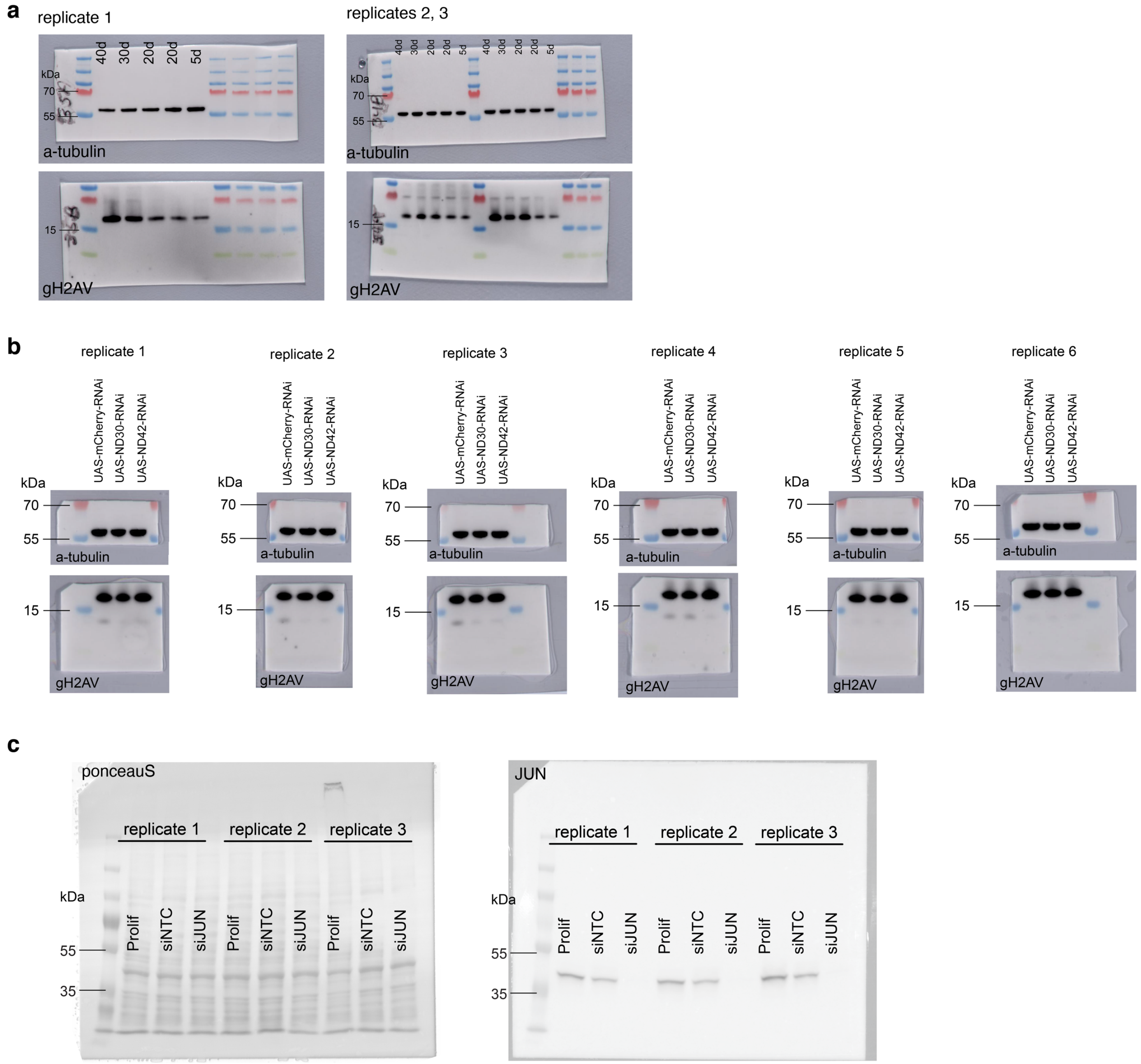

Supplementary Figure 1. Raw data for western immunoblots

a, Uncropped western immunoblots for quantification of γH2Av in aging brains. Each lane represents one biological replicate of 8-10 pooled brains. Each replicate is an independent experiment. Sample age indicated above each lane. Alpha-tubulin controls were run on the same gel; blots were cut after transfer for antibody detection. Genotype is *TRE*-dsRed.

b, Uncropped western immunoblots for quantification of γH2Av in 10d old heads with expression of neuronal-RNAi. Each lane represents one biological replicate of 8-10 pooled heads collected; each replicate is an independent experiment. Alpha-tubulin controls were run on the same gel; blots were cut after transfer for antibody detection. Genotypes are *elav*-GS>*UAS*-ND42-RNAi, *elav*-GS>*UAS*-ND30-RNAi, and *elav*-GS>*UAS*-mCherry-RNAi,.

c, Uncropped gel and immunoblot for quantification of JUN protein in cell lysate. Each replicate is an independent experiment.
